# A multi-cohort assessment of the polygenic prediction in ADHD treatment response

**DOI:** 10.1016/j.psychres.2026.116988

**Published:** 2026-01-29

**Authors:** Diego L. Rovaris, Eugenio H. Grevet, André Høberg, Pâmela F. da Cunha, Natalia Llonga, Pau Carabí-Gassol, Eduarda P. Oliveira, Cibele E. Bandeira, María Eduarda A. Tavares, María Soler Artigas, Josep Antoni Ramos-Quiroga, Christian Fadeuilhe, Montse Corrales, Vanesa Richarte, Astri J. Lundervold, Anne Halmøy, Eduardo S. Vitola, Luis A. Rohde, Marta Ribasés, Jan Haavik, Claiton H.D. Bau, Bruna S. da Silva

**Affiliations:** a Universidade de Sao Paulo, Instituto de Ciencias Biomedicas, Departamento de Fisiologia e Biofisica, São Paulo, Brazil; b ADHD Outpatient Program & Developmental Psychiatry Program, Experimental Research Center, Hospital de Clínicas de Porto Alegre, Porto Alegre, Brazil; c Department of Psychiatry and Legal Medicine, Faculty of Medicine, Universidade Federal Do Rio Grande Do Sul, Porto Alegre, Brazil; d National Institute of Developmental Psychiatry for Children and Adolescents & National Center for Research and Innovation in Child Mental Health, Sao Paulo, Brazil. Medical School Council, UniMax & UniFaj, São Paulo, Brazil; e Department of Biomedicine, University of Bergen, Bergen 5009, Norway; f Bergen Center for Brain Plasticity, Division of Psychiatry, Haukeland University Hospital, Bergen, Norway; g Psychiatric Genetics Unit, Group of Psychiatry, Mental Health and Addiction, Vall d’Hebron Research Institute (VHIR), Universitat Autònoma de Barcelona, Barcelona, Spain; h Department of Mental Health, Hospital Universitari Vall d’Hebron, Barcelona, Spain; i Biomedical Network Research Centre on Mental Health (CIBERSAM), Instituto de Salud Carlos III, Madrid, Spain; j Departament de Genètica, Microbiologia i Estadística, Facultat de Biologia, Universitat de Barcelona, Barcelona, Catalonia, Spain; k Department of Genetics and Postgraduate Program in Genetics and Molecular Biology, Universidade Federal do Rio Grande do Sul, Porto Alegre, Brazil; l Postgraduate Program in Bioscience, Federal University of Health Sciences of Porto Alegre, Rio Grande do Sul, Brazil; m Department of Psychiatry and Forensic Medicine, Universitat Autònoma de Barcelona (UAB), Barcelona, Catalonia, Spain; n Department of Biological and Medical Psychology, University of Bergen, Bergen, Norway; o Kronstad DPS, Division of Psychiatry, Haukeland University Hospital, Bergen, Norway; p Department of Clinical Medicine, University of Bergen, Bergen, Norway; q Department of Basic Health Sciences, Federal University of Health Sciences of Porto Alegre, Rio Grande do Sul, Brazil

**Keywords:** Pharmacogenomics, Treatment, Stimulants, Polygenic scores, Meta-analysis, Neurodevelopmental disorders

## Abstract

Pharmacological treatments for attention-deficit/hyperactivity disorder (ADHD) are efficacious and safe; however, substantial interindividual variability in treatment response persists, with many patients experiencing suboptimal outcomes or early discontinuation. Although genetic factors have been proposed as contributors to this variability, clinically actionable predictors remain elusive. Here, we present the first meta-analysis evaluating whether polygenic liability for ADHD and related psychiatric and behavioral-cognitive phenotypes is associated with clinically meaningful response to methylphenidate in 1000 ADHD cases from Norway, Brazil, and Spain assessed in real-world settings. Polygenic scores (PGS) for ADHD, autism, bipolar disorder, educational attainment, major depressive disorder, neuroticism, and schizophrenia were calculated separately for each cohort. Treatment response was assessed using evaluations of global clinical improvement and harmonized by categorizing individuals as responders or non-responders. Cohort-specific associations were combined using fixed-effects meta-analysis. No PGS showed a significant association with treatment response. Effect sizes were small, consistent across cohorts, and characterized by minimal between-study heterogeneity. Sensitivity analyses incorporating clinical and treatment-related covariates yielded convergent results. As the first meta-analytic evaluation of polygenic predictors evaluating clinically meaningful ADHD stimulant response, these findings delineate the current limits of PGS in pharmacogenomic applications. Rather than supporting immediate clinical utility, our results highlight key methodological and conceptual constraints, including limited sample sizes, heterogeneous outcome definitions, and the indirect nature of susceptibility-based PGS for predicting treatment response. By mapping these boundaries, this study provides a framework to recalibrate research priorities and guide the next generation of ADHD pharmacogenomic studies toward larger, harmonized, and more informative definitions of treatment response.

## 1. Introduction

Attention-Deficit/Hyperactivity Disorder (ADHD) is a common neurodevelopmental disorder, affecting approximately 5.3 % of children (Polanczyk et al., 2014) and 2.5 % of adults (Song et al., 2021). ADHD imposes substantial functional impairments across personal, social, academic, and professional domains, significantly reducing quality of life (Da Silva et al., 2023; Shaw et al., 2012). While pharmacological treatments are effective in symptom management for many individuals (Cortese et al., 2018), response varies considerably. Approximately 20–50 % of patients receiving first-line ADHD medications either fail to achieve adequate symptom control or experience detrimental adverse effects (Buitelaar and Medori, 2010; Childress and Sallee, 2014; Coghill et al., 2020). Moreover, treatment persistence remains suboptimal, with only 65 % of children and less than half of adults maintaining therapy beyond one year (Brikell et al., 2024).

Identifying factors that influence treatment response is therefore crucial for optimizing therapeutic strategies. Several clinical and sociodemographic factors have been associated with variability in ADHD treatment outcomes, including sex, perceived treatment efficacy, symptom severity, educational attainment, and a range of psychiatric and behavioral traits. These include personality traits and comorbid conditions such as schizophrenia, anxiety, major depressive, bipolar, autism spectrum, obsessive-compulsive, oppositional defiant, and substance use disorders (Brikell et al., 2021; Erkan et al., 2022; Gémes et al., 2023; Lilja et al., 2025; Retz and Retz-Junginger, 2014; Torgersen et al., 2012; Victor et al., 2009). Although pharmacogenetic studies have explored genetic contributors to response variability, results have been largely inconsistent and lack robust replication (Bonvicini et al., 2016; Capuzzi et al., 2022; Contini et al., 2013; Hegvik et al., 2016; Mick et al., 2008; Myer et al., 2018; Pagerols et al., 2018; Rovaris et al., 2014).

Additionally, genome-wide association studies (GWAS) of ADHD treatment response are constrained by the limited availability of large samples with both genome-wide and harmonized treatment outcomes data. In the absence of GWAS of treatment response itself, an alternative strategy increasingly adopted in psychiatric pharmacogenomics is to leverage polygenic scores (PGS) derived from large, well-powered GWAS of clinically and genetically correlated phenotypes to investigate predictors of treatment response. This rationale is informed by patterns observed for disease susceptibility across psychiatric disorders, where shared genetic liability underlies the high levels of clinical comorbidity (Grotzinger et al., 2026; Anttila et al., 2018). In this sense, treatment response may also be influenced by transdiagnostic genetic factors that contribute to variability in medication outcomes. For example, in major depressive disorder, higher schizophrenia PGS has been linked to poorer antidepressant response, while lower schizophrenia PGS is associated with greater treatment success (Fanelli et al., 2021). In ADHD, however, PGS applications to treatment outcomes remain scarce and have relied on indirect proxies of medication effectiveness, leaving a critical gap in the literature. To date, the largest study using PGS in this context, based on national register data from the iPSYCH2012 cohort, reported an association between treatment discontinuation and schizophrenia and bipolar disorder PGS, but did not include a clinical evaluation of treatment response (Brikell et al., 2021). Testing whether genetic associations observed with proxy outcomes translate to clinically meaningful treatment response assessed in clinical settings is therefore an important next step.

We hypothesize that genetic liability for psychiatric disorders and behavioral-cognitive traits that are epidemiologically associated with ADHD treatment outcomes, and that share genetic architecture with ADHD, also influences medication response. This study aims therefore to conduct a meta-analysis of PGS associations for ADHD and six additional major psychiatric and behavioral-cognitive phenotypes with ADHD treatment response assessed in real-world clinical settings across independent cohorts from Brazil, Norway, and Spain. Beyond addressing the associations, our study provides a comprehensive evaluation of methodological challenges in this field. By leveraging these limitations, we provide insights to guide future research efforts and to delineate the advances required in ADHD pharmacogenomics to move the field toward precision medicine in pharmacological treatment.

## 2. Methods

### 2.1. Clinical samples

We included ADHD cohorts from Brazil, Spain, and Norway, which were identified as having genome-wide genotype data and information on stimulant treatment response assessed in real-world clinical settings, with sample sizes exceeding 200 individuals. Detailed descriptions of each cohort are provided (Supplementary Methods). Briefly, the Brazilian sample included 263 adults with ADHD (subdivided into groups of European ancestry and admixed individuals) treated with methylphenidate for at least six weeks (Victor et al., 2009). The Spanish sample comprised 233 pediatric ADHD patients prescribed methylphenidate for at least eight weeks. The Norwegian sample included 503 individuals treated with methylphenidate in routine clinical care, recruited across three waves and genotyped in two batches, which were analyzed separately into two subgroups. Subjects from all cohorts met full DSM-IV or DSM-5 diagnostic criteria for ADHD. In the Brazilian and Spanish samples, treatment response was assessed using the Clinical Global Impression - Improvement (CGI-I) scale, which captures the clinician’s judgment of the patient’s global improvement, while in the Norwegian sample, clinicians or participants completed questionnaires rating treatment effect following a protocol described elsewhere (Hegvik et al., 2016). For the analyses, the outcome measure was predefined by classifying individuals into responder or non-responder groups (see Supplementary Methods for details on cohort-specific classification).

Genotyping was performed with the Infinium PsychArray-24 BeadChip and the Infinium Global Screening Array BeadChip microarrays in the Brazilian and Spanish samples, and Human OmniExpress in the Norwegian sample (more detailed description in Supplementary Methods). Quality control was applied to best-guess imputed genotypes, considering an info score > 0.8, minor allele frequency (MAF) > 0.01, individual and SNP call rate > 0.95, Hardy-Weinberg equilibrium p-value ≥ 1e-6 in controls and ≥ 1e-10 in cases.

### 2.2. Polygenic scores and association analyses

The discovery samples comprised summary statistics from GWAS of the following phenotypes: ADHD (Demontis et al., 2023), autism spectrum disorder (Grove et al., 2019), bipolar disorder (Mullins et al., 2021), major depressive disorder (Levey et al., 2021), schizophrenia (Trubetskoy et al., 2022), neuroticism (Werme et al., 2021), and educational attainment (Okbay et al., 2022). Standard quality control was performed following the tutorial for polygenic risk score analyses (Choi et al., 2020). Accordingly, the overlapping target sample was excluded from the ADHD discovery GWAS dataset.

PGS were calculated using PRS-CS, a Bayesian regression framework that applies continuous shrinkage priors to model SNP effect sizes (Ge et al., 2019). PRS-CS analyses were conducted using the default global shrinkage parameter and the linkage disequilibrium (LD) reference panel constructed from the European subset of the 1000 Genomes Project Phase 3. Posterior SNP effect size estimates were generated separately for each chromosome and subsequently concatenated to derive genome-wide scores. Individual-level polygenic scores were then computed using PLINK v1.9 (Chang et al., 2015) [www.cog-genomics.org/plink/1.9/] with the –score function. These individual generated scores were used to run all association analyses with treatment response.

Logistic regressions were performed in SPSS v21.0 software, with PGS as predictors and treatment response status (response vs non-response) as the outcome, separately for each cohort. All analyses were adjusted for age, sex, and the first five principal components (Model 1). Sensitivity analyses were performed, including additional covariates (Model 2) selected based on a predefined strategy, whereby variables were included if they were available within a given cohort and considered clinically relevant for ADHD treatment response, particularly psychiatric comorbidities and treatment-related factors. To preserve statistical power and comparability across cohorts, only covariates meeting these criteria were included. The specific covariates included in each model and cohort are detailed in the legend of Supplementary Figure S1.

### 2.3. Meta-analysis

A meta-analysis combined results from all cohorts using the *metafor R package* (https://cran.r-project.org/web/packages/metafor (Viechtbauer, 2010)), applying a fixed-effects model. The results were summarized displaying study-specific effect sizes with 95 % confidence intervals, sample sizes, mean (standard deviation) and p-values, and the pooled overall effect sizes estimates. Heterogeneity across studies was evaluated using Cochran’s Q test and I^2^ statistic, which assesses the presence and extent of variability not attributable to chance (Higgins and Thompson, 2002). Funnel plots and leave-one-out analyses were performed to detect potential outliers and assess the influence of individual studies on pooled effect estimates that could introduce heterogeneity (Viechtbauer, 2020, 2010). Statistical power for PGS was estimated using the AVENGEME R package (Palla and Dudbridge, 2015), assuming the parameters described in Table 1.

## 3. Results

In the individual cohort–level analyses, nominal associations (p < 0.05, uncorrected for multiple testing) were observed between treatment response and neuroticism PGS in the Brazilian admixed subsample in both Model 1 and Model 2, as well as between treatment response and schizophrenia PGS in the Norwegian deCODE subsample. These cohort-specific findings did not survive correction for multiple testing and were not consistent across samples. All individual-study estimates are displayed in the forest plots together with the pooled fixed-effect meta-analytic estimate (Supplementary Figure S1).

Meta-analytic results for all tested PGS, along with the corresponding forest plots, are summarized in Table 2 and Supplementary Figure S1. No significant associations were observed between any of the tested PGS and treatment response in the meta-analysis. Sensitivity analyses incorporating additional clinical and treatment-related covariates (Model 2) yielded convergent results, indicating robustness across analytic models (Table 2).

Heterogeneity was minimal, with I^2^ approaching zero in almost all analyses, and Cochran’s Q test further indicating a consistency across cohorts, with the exception of neuroticism PGS, for which moderate heterogeneity was observed, particularly in Model 2 (Table 2). Leave-one-out sensitivity analysis did not identify any single cohort that substantially affected the pooled effect estimates (Supplementary Figure S2). Funnel plots indicated no evidence of bias or between-study heterogeneity (Supplementary Figure S3). As a sensitivity analysis, we also performed the meta-analysis using random-effects (REML) models (data not shown). The model estimates results obtained were practically identical between the two models, which is consistent with the heterogeneity tests indicating low variability among the studies. The similarity of the results suggests that the studies share a common true effect, making the fixed-effects model the most appropriate approach for data interpretation.

Power calculations showed that, assuming a moderate genetic covariance (cov12 = 0.25) between genetic effect sizes in the discovery and target samples, power estimates approximate 80 % in all analysis and surpassing 95 % for several of the tested traits. However, under a more conservative assumption of lower genetic covariance (cov12 = 0.15), power estimates were notably reduced for certain traits, particularly ADHD and ASD, while remaining relatively high for others. The extent of power varied depending on the base dataset and the assumed covariance (Table 1).

## 4. Discussion

By leveraging well-powered PGS derived from GWAS for ADHD and other major psychiatric and behavioral-cognitive traits and meta-analyzing data from nearly 1000 individuals with ADHD from independent cohorts in Brazil, Norway, and Spain, our study represents one of the most comprehensive investigations to date on the genetic predictors of clinically meaningful ADHD treatment response assessed in real-world clinical settings. Among the multiple PGS-traits tested, we found no evidence of shared biological component between psychiatric and cognitive-behavioral traits and treatment response. This contrasts with previous evidence suggesting that such traits influence treatment outcomes in ADHD (Brikell et al., 2021).

PGS capture aggregated common-variant genetic liability for a given phenotype and have been widely applied to predict disease risk and related clinical outcomes. In the context of treatment response, PGS can test whether inherited liability to a disorder, or to other genetically correlated trait, contributes to variability in treatment efficacy. This approach is motivated by the hypothesis that clinical predictors of treatment response observed in practice may, at least in part, reflect shared underlying genetic architecture. Importantly, these PGS do not directly index drug-specific mechanisms, but instead reflect broader genetic backgrounds that may influence clinical presentation, comorbidity profiles, or treatment trajectories. Our findings suggest that, given current sample sizes and outcome definitions, common-variant polygenic liability explains little variance in clinically meaningful methylphenidate response, either indicating that treatment response may be more strongly influenced by factors beyond common polygenic risk for disease susceptibility, such as rare genetic variation, environmental factors, clinical heterogeneity, and treatment-related variables or methodological constraints inherent to current pharmacogenomic studies.

A pervasive challenge in the field is sample size (Bonvicini et al., 2016; Mick et al., 2008; Rovaris et al., 2014). GWAS of treatment response are currently impractical due to the lack of sufficiently powered cohorts, restricting direct gene discovery efforts while propelling the use of alternative genomic approaches, such as the combination with deep learning methods and integrative methods including expression-based prioritization and pathway enrichment (Pagerols et al., 2018; Zhao et al., 2025). As a strategy to overcome this limitation, we employed PGS derived from high-powered GWAS and combined results across multiple cohorts via meta-analysis to maximize our sample size. Despite extensive efforts to identify and harmonize datasets, making this the largest genetic study to date evaluating clinically meaningful ADHD treatment response assessed in real-world clinical settings, the cumulative target sample size remains modest, potentially limiting our ability to detect small genetic effects or subtle heterogeneity. This constraint is intrinsic to studies requiring longitudinal designs.

Our study also highlights another key limitation, which involves heterogeneity in defining and measuring treatment response across studies and the limited availability of outcome measures of treatment response. ADHD research lacks standardized definitions of treatment response, with studies relying on a wide range of approaches, including different scales to assess treatment response, variable follow-up durations, and in some cases, proxy outcomes derived from registry data (Roy et al., 2025; Shim et al., 2016). Many investigations rely on short-term scale-based symptom improvement, assessed through clinician-rated, self-reported, or informant-reported measures, but with distinct response cutoff values to define treatment response (Roy et al., 2025).

Additionally, treatment effects extend beyond core symptom reduction to broader aspects, such as improved quality of life (Bellato et al., 2025) and participant-centered outcomes (Li et al., 2024; Schneider et al., 2025), which are often overlooked in genetic studies. The multi-faceted clinical response in ADHD further complicates cross-study comparisons. In this context, clinician-rated measures offer an advantage, as they capture overall treatment benefit as perceived in clinical practice rather than small or potentially trivial symptom fluctuations. However, such measures may be more susceptible to inter-rater variability and less sensitive to subtle changes within specific symptom domains (Alavi et al., 2022; Jaeger et al., 2020).

In our meta-analysis, we harmonized treatment response measures by categorizing individuals as responders or non-responders, although outcome measures were not fully uniform across datasets. Treatment response in the Norwegian cohort was assessed using questionnaires completed by participants or clinicians, whereas the Brazilian and Spanish cohorts relied exclusively on clinician-rated CGI-I assessments. Although all outcome measures were obtained in real-world clinical settings and reflect clinically meaningful treatment response, this variability on assessment procedures may still introduce some heterogeneity. To further minimize potential sources of bias, we evaluated a range of treatment-related and clinical variables with known relevance for treatment outcomes, including medication dosage, treatment duration, use of concomitant psychiatric medications, and baseline clinical characteristics. While these variables were examined where data were available, they were not uniformly assessed across all cohorts, which limited their consistent inclusion as covariates in harmonized analyses. Notably, sensitivity analyses incorporating these additional covariates where available (Model 2) did not substantially alter the overall findings. Moreover, the absence of additional common outcome measures across cohorts and incomplete or variable information on treatment duration constrained our ability to capture the full clinical context of medication effects.

Additionally, due to the limited availability of sufficiently powered datasets, we included both pediatric and adult samples in the meta-analysis, introducing further complexities to the models. ADHD symptom presentation differs across the lifespan, with hyperactivity and impulsivity being more prominent in childhood and inattention and functional impairments persisting into adulthood (Cortese et al., 2025; Faraone et al., 2021). These developmental differences may also influence treatment response, tolerability, and dosing strategies, potentially contributing to heterogeneity in outcome measures across age groups.

Moreover, genetic ancestry differences, particularly the presence of an admixed Brazilian subgroup, could influence PGS performance, despite controlling for population stratification using principal components. However, it is important to note that the Brazilian cohort’s European ancestry component remains high, exceeding 80 % even within the admixed subgroup, as estimated by ADMIXTURE analysis (Ciochetti et al., 2025; Ramos et al., 2025). This high European ancestry proportion may mitigate some of the potential bias related to population differences between the discovery and target datasets. Still, such variability may have limited our ability to detect more robust associations. To maintain transparency, we present individual cohort results in the Supplementary Figure S1 alongside the meta-analysis forest plots, though these findings should be interpreted cautiously due to the lack of correction for multiple comparisons.

Despite these challenges, our study sought to address a fundamental issue in ADHD pharmacogenomics research, achieving the larger sample size with a meta-analysis of polygenic predictors of treatment response, using treatment outcomes assessed in real-world clinical settings. Importantly, beyond testing for associations, this study provides a systematic mapping of the current methodological and conceptual limitations of the field, including phenotype heterogeneity, limited sample sizes, and insufficient statistical power. Even in the absence of statistically robust associations, these findings are highly informative. They help delineate the current scope of polygenic approaches in ADHD pharmacogenomics, establish realistic expectations for effect sizes, and provide an empirical framework to inform the design of future studies. In this sense, our results should be viewed as an important boundary-setting contribution to ADHD pharmacogenomics, offering critical guidance for harmonization efforts, international collaboration, and the development of adequately powered investigations.

To advance in the field, collaborative efforts are crucial. Larger and harmonized datasets supported by international consortia and data-sharing initiatives will be essential to aggregate well-characterized samples, enhance statistical power, and reduce heterogeneity across studies. Establishing standardized criteria for treatment response, ideally incorporating multidimensional outcomes beyond symptom reduction, such as functional improvements, tolerability, emotional regulation, comorbidity profile, and quality of life, would not only enhance cross-study comparability but also provide a more complete picture of treatment effects (Roy et al., 2025). In parallel, large-scale consortia in the field should explicitly prioritize pharmacogenomics as a core research axis. Expanding GWAS datasets to include underrepresented ancestries and refining multi-ancestry PGS methods are also critical to improving performance in admixed populations and reducing health disparities in pharmacogenomic applications (Bruxel et al., 2025; Da Silva et al., 2026). Prioritizing these strategies is essential to advance the field toward precision medicine and to support continued investment in pharmacogenomic research on ADHD treatment response.

## 5. Conclusion

Despite the negative findings, our study clarifies why ADHD pharmacogenomics has lagged behind other disorders (Pardiñas et al., 2022; Xiong et al., 2025) and what is required for the field to become discovery-oriented. Progress in treatment resistance in schizophrenia (Pardiñas et al., 2022) and depression (Xiong et al., 2025) has been driven by (i) large-scale ascertainment through consortia and registries/biobanks, (ii) restrictive and reproducible definitions anchored to treatment trajectories, and (iii) explicit separation between susceptibility and outcome genetics, with independent validation whenever possible. Building on these principles, we propose the following priorities to move ADHD pharmacogenomics toward the level achieved in schizophrenia and major depression:

- Make pharmacogenomics a core objective within ADHD consortia, rather than treating treatment response as an opportunistic phenotype. This requires investment in harmonized treatment-outcome protocols, prospective planning for pharmacogenomic studies, and dedicated analytic workstreams designed to maximize sample size, comparability, and reproducibility across cohorts.
- Refine the collection of treatment-related data, incorporating multiple measures, such as symptom response, tolerability, discontinuation, adherence, dose optimization, comorbidity profile, use of concomitant medications, quality of life, and functional outcomes;
- Standardize definitions of treatment response to enable portability across studies and countries. Following successful models in treatment resistance research, prioritize reproducible algorithmic phenotypes derived from routine care, and use restrictive definitions when the goal is increased homogeneity.
- Scale through registries and biobanks, then validate in deeply phenotyped clinical cohorts. Use health-record–based cohorts to achieve order-of-magnitude sample size gains, and complement them with smaller but richer clinical samples for calibration, mechanistic interpretation, and external validation.
- Run GWAS of treatment outcomes directly to avoid relying exclusively on susceptibility-based PGS. The most informative polygenic predictors for response will ultimately come from GWAS of response itself; until then, susceptibility PGS should be framed as indirect tests of background liability rather than drug-specific biology.
- Expand beyond common variants to include rare and structural variation, as treatment resistance phenotypes may be influenced by rare and larger-effect variation.
- Prioritize ancestry diversity and methods that work in admixed populations to increase representation of underrepresented ancestries in both discovery and target datasets, so pharmacogenomic applications do not widen disparities.
- Adopt an open-science infrastructure that makes meta-analysis effortless. Share harmonized phenotype codebooks, analysis scripts, and summary statistics whenever possible, enabling continuous accumulation of evidence and rapid replication.

Together, these steps provide a realistic path from the current underpowered and heterogeneous landscape to a mature, scalable framework where ADHD treatment response becomes a tractable genetic outcome and a credible target for precision psychiatry.

## Supplementary Material

1

2

3

4

5

Supplementary material associated with this article can be found, in the online version, at doi:10.1016/j.psychres.2026.116988.

## Figures and Tables

**Table 1:** Power estimates for the polygenic score meta-analysis, including parameter assumptions for each discovery dataset, calculated using the AVENGEME R package.

Discovery dataset	Reference	vg1	N cases	N controls	N total	Prev.^b^	π0	Pupper	Power for cov12 = 0.25	Power for cov12 = 0.15
ADHD^a^	Demontis et al., 2023.	0.14	38,691	186,843	225,534	0.05	0.99	c(0, 1)	0.846	0.430
ASD	Grove et al., 2019.	0.118	18,381	27,969	46,35	0.01	0.99	c(0, 1)	0.764	0.360
BD	Mullins, 2021.	0.186	41,917	371,549	413,466	0.02	0.99	c(0, 1)	0.984	0.685
EA	Okbay et al., 2022.	0.12	NA	NA	3037,499	NA	0.99	c(0, 1)	1	1
MDD	Levey et al., 2021.	0.113	340,591	813,676	1154,267	0.15	0.99	c(0, 1)	0.999	0.926
NEU	Werme et al., 2021.	0.12	NA	NA	313,339	NA	0.99	c(0,1)	0.999	0.882
SCZ	Trubetskoy et al., 2022.	0.24	76,755	243,649	320,404	0.03	0.99	c(0, 1)	0.997	0.816

ADHD, attention-deficit/hyperactivity disorder; ASD, autism spectrum disorder; BD, bipolar disorder; EA, educational attainment; MDD, major depressive disorder; NEU, neuroticism; SCZ, schizophrenia; N, sample size; Prev., prevalence; vg1, proportion of variance explained by genetic effects in training sample or single nucleotide polymorphism heritability (h^2^SNP); cov12, covariance between genetic effect sizes in the two samples; π0, proportion of markers with no effect on the training trait; pupper, vector of p-value thresholds for selecting markers from training sample, where first element is the lower bound and second element is the upper bound; NA, not applicable.

a excluded Bergen sample to avoid overlap between discovery and target samples;

b Disease prevalence in the population.

**Table 2:** Meta-analysis results of the associations between polygenic scores for psychiatric and cognitive-behavioral traits and treatment response.

Analysis model ADHD^a^	OR	Lower CI (95 %)	Upper CI (95 %)	P-value	I2 (%)	H2	Q	Q(pval)
*Model 1* ^b^	1.08	0.90	1.29	0.9475	0	0.18	0.7307	0.9475
*Model 2* ^c^	1.08	0.88	1.31	0.4666	0	0.60	2.4049	0.6617
**ASD**								
*Model 1* ^b^	0.94	0.77	1.15	0.5630	0	0.65	2.6151	0.6241
*Model 2* ^c^	0.99	0.78	0.1.24	0.9058	0	0.46	1.8482	0.7637
**BD**								
*Model 1* ^b^	0.94	0.74	1.20	0.6445	0	0.29	1.1654	0.8838
*Model 2* ^c^	0.94	0.71	1.25	0.6727	0	0.56	2.2460	0.6906
**EA**								
*Model 1* ^b^	1.04	0.84	1.29	0.7094	43.0	1.75	7.0144	0.1351
*Model 2* ^c^	1.17	0.91	1.49	0.2186	0	0.81	3.2404	0.5184
**MDD**								
*Model 1* ^b^	1.05	0.85	1.30	0.6538	0	0.49	1.9430	0.7462
*Model 2* ^c^	1.02	0.79	1.30	0.9038	0	0.34	1.3551	0.8520
**NEU**								
*Model 1* ^b^	0.99	0.82	1.19	0.8888	38.5	1.63	6.5006	0.1648
*Model 2* ^c^	0.89	0.72	1.10	0.2809	57.9	2.38	9.5108	0.0495
**SCZ**								
*Model 1* ^b^	0.89	0.74	1.07	0.2170	0	0.94	3.7686	0.4382
*Model 2* ^c^	0.95	0.78	1.17	0.6436	0	0.54	2.1612	0.7061

ADHD, attention-deficit/hyperactivity disorder; ASD, autism spectrum disorder; BD, bipolar disorder; EA, educational attainment; MDD, major depressive disorder; NEU, neuroticism; SCZ, schizophrenia; CI, confidence interval; OR, odds ratio; Q, Cochran’s Q test for heterogeneity; Q(pval), p-value for Q; I2 statistic, calculated as total heterogeneity/total variability. H2 statistic, calculated as total variability/sampling variability.

a excluded Bergen sample to avoid overlap between discovery and target samples;

b Adjusted for age, sex and first five principal components;

c Adjusted for age, sex, the first five principal components, and additional covariates selected on a cohort-specific basis according to their availability and clinical relevance for ADHD treatment response; the specific covariates included in each model and cohort are detailed in the legend of Supplementary Figure S1.
