# Supplementary material for: A multi-cohort assessment of the polygenic prediction in ADHD treatment response
Source: Psychiatry Res. Author manuscript; Available in PMC 2026 Jul 7. (PMC13340436; doi:10.1016/j.psychres.2026.116988)
Supplement: 1 [file NIHMS2190217-supplement-1.docx]

**A multi-cohort assessment of the polygenic prediction in ADHD treatment response**

**SUPPLEMENTARY METHODS**

*Brazilian sample*

This sample comprised 263 adults with ADHD treated with immediate-release or long-acting methylphenidate. Individuals with ADHD were ascertained from the ADHD Outpatient Program in the adult division at *Hospital de Clínicas de Porto Alegre* (HCPA), Brazil. More detailed diagnostic procedures for ADHD and comorbidities have been described elsewhere (Grevet et al., 2005; Victor et al., 2014). All participants signed an informed consent form previously approved by the institutional review board of the hospital (No. 00000921). The study was carried out in accordance with the World Medical Association Declaration of Helsinki.

The treatment protocol was based on procedures previously established to investigate clinical predictors of methylphenidate treatment response (Victor et al., 2009). After stabilization of comorbidities and reevaluation to confirm maintenance of ADHD diagnosis, those eligible for inclusion in treatment protocol received methylphenidate with doses increasing weekly until symptom control or occurrence of limiting adverse effects. The standard initial daily dose was 10 mg, and the post-treatment evaluation was assessed after 45 to 60 days of treatment on average. The treatment response was evaluated based on clinical judgment of improvement considering the Clinical Global Impression - Improvement (CGI-I) scale. The CGI-I scale is composed of scores ranging from 1 (very much improved) to 7 (very much worse). The outcome measure considered the classification of individuals into groups of responders (CGI-I <= 2) and non-responders (CGI-I >=3). Treatment response was assessed by the same clinician (total of three evaluators involved) who prescribed the medication. They are all trained and experienced clinicians, reflecting real-world clinical practice and minimizing different evaluator-related variability.

Genotyping was performed on the Infinium PsychArray-24 BeadChip platform (PsychChip, Illumina, San Diego, CA, USA), Broad Institute of Harvard and MIT (Cambridge, MA, USA) and the Infinium™ Global Screening Array (GSA, Illumina, San Diego, CA, USA) BeadChip microarray (versions 1.0+MD and 3.0+MD). Quality control (QC) and principal components analysis (PCA) were implemented using the default parameter on RICOPILI (Lam et al., 2020) pipeline (<https://sites.google.com/a/broadinstitute.org/ricopili/home>). The Michigan Imputation Server (Das et al., 2016) using Minimac4 and the TOPMed reference panel was used for phasing and genotype imputation. After post imputation QC performed in Plink2 (Chang et al., 2015), the combined dataset consisted of 5,751,409 SNPs with info score > 0.8, a genotype call probability > 0.95, a minor allele frequency > 0.01 and a missing rate <0.95 for 1,660 individuals. Multidimensional Scaling (MDS) (Marees et al., 2018) and maximum likelihood estimation (ADMIXTURE) (Alexander et al., 2009) were performed to access the degree of admixture and classify groups of individuals into European ancestry (EUR, those with MDS components 1 and 2 values corresponding to the range observed for the 1,000 genomes European population; n = 136; European component ~90%) and (ADMIX, those outside this range; n = 127; European component ~80%) (data not shown; for more detailed information see (Ramos et al., 2025)). Response rates for these groups were 78% and 77%, respectively.

*Norwegian sample*

This sample comprised 503 adults with ADHD treated with immediate-release or long-acting methylphenidate. Individuals with ADHD were ascertained from the 'ADHD in Norwegian Adults' (ANA) project. The project recruited patients from a national registry of adults diagnosed with ADHD between 1997 and 2005 and psychiatric outpatient clinics between 2005 and 2020 (Halmøy et al., 2010). For details on recruitment, sociodemographic characteristics, and clinical features, see Halmøy et al. 2010. All participants provided a signed informed consent.

Treatment response was assessed using questionnaires completed by participants or clinicians (two evaluators were involved), evaluating the efficacy of medication prescribed by the patients’ treating adult psychiatrists. The questionnaires assessed whether the effect of methylphenidate was “Very Good”, “Good” or “No Effect”. Participants with the “No Effect” response were considered non-responders, while participants with other responses were considered responders. For details on questionnaires and response rating, see Hegvik et al., 2016).

DNA from saliva samples were genotyped in two batches: one at the Broad Institute (Boston, MA, USA), genotyped on the Human OmniExpress-12v1-1_B (Illumina, San Diego, CA, USA) platform, and one at deCODE (Reykjavik, Iceland), genotyped on the Human OmniExpress-12v1_H (Illumina, San Diego, CA, USA) platform. Genotypes were assigned according to the standard Illumina protocol in GenomeStudio software, version V2011.1. Quality control and imputation were performed using Ricopili (Lam et al., 2020).

*Spanish sample*

This sample comprised 233 pediatric patients who were required to satisfy full DSM-IV criteria for ADHD, be under 18 years of age, Spanish of Caucasian origin and have never received MPH treatment. Patients with an IQ below 70 or having pervasive developmental disorders were not eligible for the investigation. Additional exclusion criteria included schizophrenia or other psychotic disorders; adoption; sexual or physical abuse; birth weight <1.5 kg; any significant neurological or systemic disease that might explain ADHD symptoms; and clinical contra-indication to MPH. The study was approved by the Ethics Committee of the Hospital Universitari Vall d’Hebron and all methods were performed in accordance with the relevant guidelines and regulations. Written informed consent was obtained from parents/caregivers.

The treatment response was evaluated based on clinical judgment of improvement considering the CGI-I scale, and the treatment outcome measure was defined following the same criteria as the Brazilian sample. Treatment response was assessed by the same clinicians who prescribed the medication (one evaluator involved).

Genomic DNA was isolated from whole blood using the salting-out method. Genotyping was conducted in three separate waves, using two different arrays: 171 individuals with Illumina Infinium PsychChip v1.0 array and 62 individuals with Infinium™ Global Screening Array-24 v2.0. Pre-imputation quality control was performed separately for each wave with Plink2 (Chang et al., 2015) [https://www.cog-genomics.org/plink/2.0/]. These steps included filtering both individuals and variants based on the following parameters: variant call rate >0.95 (before individual filtering), individual call rate >0.98, autosomal heterozygosity deviation (|Fhet|<0.2), variant call rate >0.98 (after individual filtering), SNP Hardy-Weinberg equilibrium (HWE; P>1e−10) and minor allele frequency (MAF)>0.01. Subjects included were European and ancestry outliers were identified by principal component analysis (PCA) using Plink2 (Chang et al., 2015) and the mixed ancestry 1000G reference panel (Auton et al., 2015). Ancestry outliers were excluded if their principal component (PC) values for PC1 or PC2 were greater than 1 standard deviation from the mean-centering point for our sample, considering each GWAS wave separately. Imputation was done with McCarthy tools, for data preparation, and the Michigan Imputation Server (Das et al., 2016), using the Haplotype Reference Consortium (HRC Version r1.1 2016) reference panel (GRCh37/hg19). After imputation, genotype hard calls were obtained from imputed dosages using Plink2, which by default applies a threshold (--hard-call-threshold 0.1) to decide whether to assign a genotype or a missing value. Then, we applied post-imputation quality control filters, keeping only SNPs with an imputation INFO score >0.8, minor allele frequency (MAF) >0.01 and SNP call rate >0.95.

Then, samples from the different GWAS waves were merged and only SNPs present in all of them, with MAF >0.01, SNP call rate >0.95 and SNP Hardy-Weinberg equilibrium (HWE; P>1e−6) in the overall sample were considered for subsequent analyses. Related and duplicated samples were identified after merging the imputed data from all genotyping waves, using the “KING-robust kinship estimator” analysis Plink2 (Chang et al., 2015), and one individual was excluded from each pair of subjects with kinship coefficient >0.0442.

**REFERENCES**

Alexander, D.H., Novembre, J., Lange, K., 2009. Fast model-based estimation of ancestry in unrelated individuals. Genome Res. 19, 1655–1664. https://doi.org/10.1101/gr.094052.109

Auton, A., Abecasis, G.R., Steering committee, The 1000 Genomes Project Consortium, Altshuler, D.M., Durbin, R.M., Abecasis, G.R., Bentley, D.R., Chakravarti, A., Clark, A.G., Donnelly, P., Eichler, E.E., Flicek, P., Gabriel, S.B., Gibbs, R.A., Green, E.D., Hurles, M.E., Knoppers, B.M., Korbel, J.O., Lander, E.S., Lee, C., Lehrach, H., Mardis, E.R., Marth, G.T., McVean, G.A., Nickerson, D.A., Schmidt, J.P., Sherry, S.T., Wang, J., Wilson, R.K., Production group, Baylor College of Medicine, Gibbs, R.A., Boerwinkle, E., Doddapaneni, H., Han, Y., Korchina, V., Kovar, C., Lee, S., Muzny, D., Reid, J.G., Zhu, Y., BGI-Shenzhen, Wang, J., Chang, Y., Feng, Q., Fang, X., Guo, X., Jian, M., Jiang, H., Jin, X., Lan, T., Li, G., Li, J., Li, Yingrui, Liu, S., Liu, Xiao, Lu, Y., Ma, X., Tang, M., Wang, B., Wang, G., Wu, H., Wu, R., Xu, X., Yin, Y., Zhang, D., Zhang, W., Zhao, J., Zhao, M., Zheng, X., Broad Institute of MIT and Harvard, Lander, E.S., Altshuler, D.M., Gabriel, S.B., Gupta, N., Coriell Institute for Medical Research, Gharani, N., Toji, L.H., Gerry, N.P., Resch, A.M., European Molecular Biology Laboratory, European Bioinformatics Institute, Flicek, P., Barker, J., Clarke, L., Gil, L., Hunt, S.E., Kelman, G., Kulesha, E., Leinonen, R., McLaren, W.M., Radhakrishnan, R., Roa, A., Smirnov, D., Smith, R.E., Streeter, I., Thormann, A., Toneva, I., Vaughan, B., Zheng-Bradley, X., Illumina, Bentley, D.R., Grocock, R., Humphray, S., James, T., Kingsbury, Z., Max Planck Institute for Molecular Genetics, Lehrach, H., Sudbrak, R., Albrecht, M.W., Amstislavskiy, V.S., Borodina, T.A., Lienhard, M., Mertes, F., Sultan, M., Timmermann, B., Yaspo, M.-L., McDonnell Genome Institute at Washington University, Mardis, E.R., Wilson, R.K., Fulton, L., Fulton, R., US National Institutes of Health, Sherry, S.T., Ananiev, V., Belaia, Z., Beloslyudtsev, D., Bouk, N., Chen, C., Church, D., Cohen, R., Cook, C., Garner, J., Hefferon, T., Kimelman, M., Liu, C., Lopez, J., Meric, P., O’Sullivan, C., Ostapchuk, Y., Phan, L., Ponomarov, S., Schneider, V., Shekhtman, E., Sirotkin, K., Slotta, D., Zhang, H., University of Oxford, McVean, G.A., Wellcome Trust Sanger Institute, Durbin, R.M., Balasubramaniam, S., Burton, J., Danecek, P., Keane, T.M., Kolb-Kokocinski, A., McCarthy, S., Stalker, J., Quail, M., Analysis group, Affymetrix, Schmidt, J.P., Davies, C.J., Gollub, J., Webster, T., Wong, B., Zhan, Y., Albert Einstein College of Medicine, Auton, A., Campbell, C.L., Kong, Y., Marcketta, A., Baylor College of Medicine, Gibbs, R.A., Yu, F., Antunes, L., Bainbridge, M., Muzny, D., Sabo, A., Huang, Z., BGI-Shenzhen, Wang, J., Coin, L.J.M., Fang, L., Guo, X., Jin, X., Li, G., Li, Q., Li, Yingrui, Li, Z., Lin, H., Liu, B., Luo, R., Shao, H., Xie, Y., Ye, C., Yu, C., Zhang, F., Zheng, H., Zhu, H., Bilkent University, Alkan, C., Dal, E., Kahveci, F., Boston College, Marth, G.T., Garrison, E.P., Kural, D., Lee, W.-P., Fung Leong, W., Stromberg, M., Ward, A.N., Wu, J., Zhang, M., Broad Institute of MIT and Harvard, Daly, M.J., DePristo, M.A., Handsaker, R.E., Altshuler, D.M., Banks, E., Bhatia, G., Del Angel, G., Gabriel, S.B., Genovese, G., Gupta, N., Li, H., Kashin, S., Lander, E.S., McCarroll, S.A., Nemesh, J.C., Poplin, R.E., Cold Spring Harbor Laboratory, Yoon, S.C., Lihm, J., Makarov, V., Cornell University, Clark, A.G., Gottipati, S., Keinan, A., Rodriguez-Flores, J.L., European Molecular Biology Laboratory, Korbel, J.O., Rausch, T., Fritz, M.H., Stütz, A.M., European Molecular Biology Laboratory, European Bioinformatics Institute, Flicek, P., Beal, K., Clarke, L., Datta, A., Herrero, J., McLaren, W.M., Ritchie, G.R.S., Smith, R.E., Zerbino, D., Zheng-Bradley, X., Harvard University, Sabeti, P.C., Shlyakhter, I., Schaffner, S.F., Vitti, J., Human Gene Mutation Database, Cooper, D.N., Ball, E.V., Stenson, P.D., Illumina, Bentley, D.R., Barnes, B., Bauer, M., Keira Cheetham, R., Cox, A., Eberle, M., Humphray, S., Kahn, S., Murray, L., Peden, J., Shaw, R., Icahn School of Medicine at Mount Sinai, Kenny, E.E., Louisiana State University, Batzer, M.A., Konkel, M.K., Walker, J.A., Massachusetts General Hospital, MacArthur, D.G., Lek, M., Max Planck Institute for Molecular Genetics, Sudbrak, R., Amstislavskiy, V.S., Herwig, R., McDonnell Genome Institute at Washington University, Mardis, E.R., Ding, L., Koboldt, D.C., Larson, D., Ye, Kai, McGill University, Gravel, S., National Eye Institute, NIH, Swaroop, A., Chew, E., New York Genome Center, Lappalainen, T., Erlich, Y., Gymrek, M., Frederick Willems, T., Ontario Institute for Cancer Research, Simpson, J.T., Pennsylvania State University, Shriver, M.D., Rutgers Cancer Institute of New Jersey, Rosenfeld, J.A., Stanford University, Bustamante, C.D., Montgomery, S.B., De La Vega, F.M., Byrnes, J.K., Carroll, A.W., DeGorter, M.K., Lacroute, P., Maples, B.K., Martin, A.R., Moreno-Estrada, A., Shringarpure, S.S., Zakharia, F., Tel-Aviv University, Halperin, E., Baran, Y., The Jackson Laboratory for Genomic Medicine, Lee, C., Cerveira, E., Hwang, J., Malhotra, A., Plewczynski, D., Radew, K., Romanovitch, M., Zhang, C., Thermo Fisher Scientific, Hyland, F.C.L., Translational Genomics Research Institute, Craig, D.W., Christoforides, A., Homer, N., Izatt, T., Kurdoglu, A.A., Sinari, S.A., Squire, K., US National Institutes of Health, Sherry, S.T., Xiao, C., University of California, San Diego, Sebat, J., Antaki, D., Gujral, M., Noor, A., Ye, Kenny, University of California, San Francisco, Burchard, E.G., Hernandez, R.D., Gignoux, C.R., University of California, Santa Cruz, Haussler, D., Katzman, S.J., James Kent, W., University of Chicago, Howie, B., University College London, Ruiz-Linares, A., University of Geneva, Dermitzakis, E.T., University of Maryland School of Medicine, Devine, S.E., University of Michigan, Abecasis, G.R., Min Kang, H., Kidd, J.M., Blackwell, T., Caron, S., Chen, W., Emery, S., Fritsche, L., Fuchsberger, C., Jun, G., Li, B., Lyons, R., Scheller, C., Sidore, C., Song, S., Sliwerska, E., Taliun, D., Tan, A., Welch, R., Kate Wing, M., Zhan, X., University of Montréal, Awadalla, P., Hodgkinson, A., University of North Carolina at Chapel Hill, Li, Yun, University of North Carolina at Charlotte, Shi, X., Quitadamo, A., University of Oxford, Lunter, G., McVean, G.A., Marchini, J.L., Myers, S., Churchhouse, C., Delaneau, O., Gupta-Hinch, A., Kretzschmar, W., Iqbal, Z., Mathieson, I., Menelaou, A., Rimmer, A., Xifara, D.K., University of Puerto Rico, Oleksyk, T.K., University of Texas Health Sciences Center at Houston, Fu, Yunxin, Liu, Xiaoming, Xiong, M., University of Utah, Jorde, L., Witherspoon, D., Xing, J., University of Washington, Eichler, E.E., Browning, B.L., Browning, S.R., Hormozdiari, F., Sudmant, P.H., Weill Cornell Medical College, Khurana, E., Wellcome Trust Sanger Institute, Durbin, R.M., Hurles, M.E., Tyler-Smith, C., Albers, C.A., Ayub, Q., Balasubramaniam, S., Chen, Y., Colonna, V., Danecek, P., Jostins, L., Keane, T.M., McCarthy, S., Walter, K., Xue, Y., Yale University, Gerstein, M.B., Abyzov, A., Balasubramanian, S., Chen, J., Clarke, D., Fu, Yao, Harmanci, A.O., Jin, M., Lee, D., Liu, J., Jasmine Mu, X., Zhang, J., Zhang, Yan, Structural variation group, BGI-Shenzhen, Li, Yingrui, Luo, R., Zhu, H., Bilkent University, Alkan, C., Dal, E., Kahveci, F., Boston College, Marth, G.T., Garrison, E.P., Kural, D., Lee, W.-P., Ward, A.N., Wu, J., Zhang, M., Broad Institute of MIT and Harvard, McCarroll, S.A., Handsaker, R.E., Altshuler, D.M., Banks, E., Del Angel, G., Genovese, G., Hartl, C., Li, H., Kashin, S., Nemesh, J.C., Shakir, K., Cold Spring Harbor Laboratory, Yoon, S.C., Lihm, J., Makarov, V., Cornell University, Degenhardt, J., European Molecular Biology Laboratory, Korbel, J.O., Fritz, M.H., Meiers, S., Raeder, B., Rausch, T., Stütz, A.M., European Molecular Biology Laboratory, European Bioinformatics Institute, Flicek, P., Paolo Casale, F., Clarke, L., Smith, R.E., Stegle, O., Zheng-Bradley, X., Illumina, Bentley, D.R., Barnes, B., Keira Cheetham, R., Eberle, M., Humphray, S., Kahn, S., Murray, L., Shaw, R., Leiden University Medical Center, Lameijer, E.-W., Louisiana State University, Batzer, M.A., Konkel, M.K., Walker, J.A., McDonnell Genome Institute at Washington University, Ding, L., Hall, I., Ye, Kai, Stanford University, Lacroute, P., The Jackson Laboratory for Genomic Medicine, Lee, C., Cerveira, E., Malhotra, A., Hwang, J., Plewczynski, D., Radew, K., Romanovitch, M., Zhang, C., Translational Genomics Research Institute, Craig, D.W., Homer, N., US National Institutes of Health, Church, D., Xiao, C., University of California, San Diego, Sebat, J., Antaki, D., Bafna, V., Michaelson, J., Ye, Kenny, University of Maryland School of Medicine, Devine, S.E., Gardner, E.J., University of Michigan, Abecasis, G.R., Kidd, J.M., Mills, R.E., Dayama, G., Emery, S., Jun, G., University of North Carolina at Charlotte, Shi, X., Quitadamo, A., University of Oxford, Lunter, G., McVean, G.A., University of Texas MD Anderson Cancer Center, Chen, K., Fan, X., Chong, Z., Chen, T., University of Utah, Witherspoon, D., Xing, J., University of Washington, Eichler, E.E., Chaisson, M.J., Hormozdiari, F., Huddleston, J., Malig, M., Nelson, B.J., Sudmant, P.H., Vanderbilt University School of Medicine, Parrish, N.F., Weill Cornell Medical College, Khurana, E., Wellcome Trust Sanger Institute, Hurles, M.E., Blackburne, B., Lindsay, S.J., Ning, Z., Walter, K., Zhang, Yujun, Yale University, Gerstein, M.B., Abyzov, A., Chen, J., Clarke, D., Lam, H., Jasmine Mu, X., Sisu, C., Zhang, J., Zhang, Yan, Exome group, Baylor College of Medicine, Gibbs, R.A., Yu, F., Bainbridge, M., Challis, D., Evani, U.S., Kovar, C., Lu, J., Muzny, D., Nagaswamy, U., Reid, J.G., Sabo, A., Yu, J., BGI-Shenzhen, Guo, X., Li, W., Li, Yingrui, Wu, R., Boston College, Marth, G.T., Garrison, E.P., Fung Leong, W., Ward, A.N., Broad Institute of MIT and Harvard, Del Angel, G., DePristo, M.A., Gabriel, S.B., Gupta, N., Hartl, C., Poplin, R.E., Cornell University, Clark, A.G., Rodriguez-Flores, J.L., European Molecular Biology Laboratory, European Bioinformatics Institute, Flicek, P., Clarke, L., Smith, R.E., Zheng-Bradley, X., Massachusetts General Hospital, MacArthur, D.G., McDonnell Genome Institute at Washington University, Mardis, E.R., Fulton, R., Koboldt, D.C., McGill University, Gravel, S., Stanford University, Bustamante, C.D., Translational Genomics Research Institute, Craig, D.W., Christoforides, A., Homer, N., Izatt, T., US National Institutes of Health, Sherry, S.T., Xiao, C., University of Geneva, Dermitzakis, E.T., University of Michigan, Abecasis, G.R., Min Kang, H., University of Oxford, McVean, G.A., Yale University, Gerstein, M.B., Balasubramanian, S., Habegger, L., Functional interpretation group, Cornell University, Yu, H., European Molecular Biology Laboratory, European Bioinformatics Institute, Flicek, P., Clarke, L., Cunningham, F., Dunham, I., Zerbino, D., Zheng-Bradley, X., Harvard University, Lage, K., Berg Jespersen, J., Horn, H., Stanford University, Montgomery, S.B., DeGorter, M.K., Weill Cornell Medical College, Khurana, E., Wellcome Trust Sanger Institute, Tyler-Smith, C., Chen, Y., Colonna, V., Xue, Y., Yale University, Gerstein, M.B., Balasubramanian, S., Fu, Yao, Kim, D., Chromosome Y group, Albert Einstein College of Medicine, Auton, A., Marcketta, A., American Museum of Natural History, Desalle, R., Narechania, A., Arizona State University, Wilson Sayres, M.A., Boston College, Garrison, E.P., Broad Institute of MIT and Harvard, Handsaker, R.E., Kashin, S., McCarroll, S.A., Cornell University, Rodriguez-Flores, J.L., European Molecular Biology Laboratory, European Bioinformatics Institute, Flicek, P., Clarke, L., Zheng-Bradley, X., New York Genome Center, Erlich, Y., Gymrek, M., Frederick Willems, T., Stanford University, Bustamante, C.D., Mendez, F.L., David Poznik, G., Underhill, P.A., The Jackson Laboratory for Genomic Medicine, Lee, C., Cerveira, E., Malhotra, A., Romanovitch, M., Zhang, C., University of Michigan, Abecasis, G.R., University of Queensland, Coin, L., Shao, H., Virginia Bioinformatics Institute, Mittelman, D., Wellcome Trust Sanger Institute, Tyler-Smith, C., Ayub, Q., Banerjee, R., Cerezo, M., Chen, Y., Fitzgerald, T.W., Louzada, S., Massaia, A., McCarthy, S., Ritchie, G.R., Xue, Y., Yang, F., Data coordination center group, Baylor College of Medicine, Gibbs, R.A., Kovar, C., Kalra, D., Hale, W., Muzny, D., Reid, J.G., BGI-Shenzhen, Wang, J., Dan, X., Guo, X., Li, G., Li, Yingrui, Ye, C., Zheng, X., Broad Institute of MIT and Harvard, Altshuler, D.M., European Molecular Biology Laboratory, European Bioinformatics Institute, Flicek, P., Clarke, L., Zheng-Bradley, X., Illumina, Bentley, D.R., Cox, A., Humphray, S., Kahn, S., Max Planck Institute for Molecular Genetics, Sudbrak, R., Albrecht, M.W., Lienhard, M., McDonnell Genome Institute at Washington University, Larson, D., Translational Genomics Research Institute, Craig, D.W., Izatt, T., Kurdoglu, A.A., US National Institutes of Health, Sherry, S.T., Xiao, C., University of California, Santa Cruz, Haussler, D., University of Michigan, Abecasis, G.R., University of Oxford, McVean, G.A., Wellcome Trust Sanger Institute, Durbin, R.M., Balasubramaniam, S., Keane, T.M., McCarthy, S., Stalker, J., Samples and ELSI group, Chakravarti, A., Knoppers, B.M., Abecasis, G.R., Barnes, K.C., Beiswanger, C., Burchard, E.G., Bustamante, C.D., Cai, H., Cao, H., Durbin, R.M., Gerry, N.P., Gharani, N., Gibbs, R.A., Gignoux, C.R., Gravel, S., Henn, B., Jones, D., Jorde, L., Kaye, J.S., Keinan, A., Kent, A., Kerasidou, A., Li, Yingrui, Mathias, R., McVean, G.A., Moreno-Estrada, A., Ossorio, P.N., Parker, M., Resch, A.M., Rotimi, C.N., Royal, C.D., Sandoval, K., Su, Y., Sudbrak, R., Tian, Z., Tishkoff, S., Toji, L.H., Tyler-Smith, C., Via, M., Wang, Y., Yang, H., Yang, L., Zhu, J., Sample collection, British from England and Scotland (GBR), Bodmer, W., Colombians in Medellín, Colombia (CLM), Bedoya, G., Ruiz-Linares, A., Han Chinese South (CHS), Cai, Z., Gao, Y., Chu, J., Finnish in Finland (FIN), Peltonen, L., Iberian Populations in Spain (IBS), Garcia-Montero, A., Orfao, A., Puerto Ricans in Puerto Rico (PUR), Dutil, J., Martinez-Cruzado, J.C., Oleksyk, T.K., African Caribbean in Barbados (ACB), Barnes, K.C., Mathias, R.A., Hennis, A., Watson, H., McKenzie, C., Bengali in Bangladesh (BEB), Qadri, F., LaRocque, R., Sabeti, P.C., Chinese Dai in Xishuangbanna, China (CDX), Zhu, J., Deng, X., Esan in Nigeria (ESN), Sabeti, P.C., Asogun, D., Folarin, O., Happi, C., Omoniwa, O., Stremlau, M., Tariyal, R., Gambian in Western Division – Mandinka (GWD), Jallow, M., Sisay Joof, F., Corrah, T., Rockett, K., Kwiatkowski, D., Indian Telugu in the UK (ITU) and Sri Lankan Tamil in the UK (STU), Kooner, J., Kinh in Ho Chi Minh City, Vietnam (KHV), Tịnh Hiê`n, T., Dunstan, S.J., Thuy Hang, N., Mende in Sierra Leone (MSL), Fonnie, R., Garry, R., Kanneh, L., Moses, L., Sabeti, P.C., Schieffelin, J., Grant, D.S., Peruvian in Lima, Peru (PEL), Gallo, C., Poletti, G., Punjabi in Lahore, Pakistan (PJL), Saleheen, D., Rasheed, A., Scientific management, Brooks, L.D., Felsenfeld, A.L., McEwen, J.E., Vaydylevich, Y., Green, E.D., Duncanson, A., Dunn, M., Schloss, J.A., Wang, J., Yang, H., Writing group, Auton, A., Brooks, L.D., Durbin, R.M., Garrison, E.P., Min Kang, H., Korbel, J.O., Marchini, J.L., McCarthy, S., McVean, G.A., Abecasis, G.R., 2015. A global reference for human genetic variation. Nature 526, 68–74. https://doi.org/10.1038/nature15393

Chang, C.C., Chow, C.C., Tellier, L.C., Vattikuti, S., Purcell, S.M., Lee, J.J., 2015. Second-generation PLINK: rising to the challenge of larger and richer datasets. Gigascience 4, s13742-015-0047–8. https://doi.org/10.1186/s13742-015-0047-8

Das, S., Forer, L., Schönherr, S., Sidore, C., Locke, A.E., Kwong, A., Vrieze, S.I., Chew, E.Y., Levy, S., McGue, M., Schlessinger, D., Stambolian, D., Loh, P.-R., Iacono, W.G., Swaroop, A., Scott, L.J., Cucca, F., Kronenberg, F., Boehnke, M., Abecasis, G.R., Fuchsberger, C., 2016. Next-generation genotype imputation service and methods. Nat Genet 48, 1284–1287. https://doi.org/10.1038/ng.3656

Grevet, E.H., Bau, C.H.D., Salgado, C.A.I., Ficher, A., Victor, M.M., Garcia, C., Sousa, N.O.D., Nerung, L., Belmonte-de-Abreu, P., 2005. Concordância entre observadores para o diagnóstico em adultos do trantorno de déficit de atenção/hiperatividade e transtorno de oposição desafiante utilizando o K-SADS-E. Arq. Neuro-Psiquiatr. 63, 307–310. https://doi.org/10.1590/S0004-282X2005000200019

Halmøy, A., Halleland, H., Dramsdahl, M., Bergsholm, P., Fasmer, O.B., Haavik, J., 2010. Bipolar Symptoms in Adult Attention-Deficit/Hyperactivity Disorder: A Cross-Sectional Study of 510 Clinically Diagnosed Patients and 417 Population-Based Controls. J. Clin. Psychiatry 71, 48–57. https://doi.org/10.4088/JCP.08m04722ora

Hegvik, T.-A., Jacobsen, K.K., Fredriksen, M., Zayats, T., Haavik, J., 2016. A candidate gene investigation of methylphenidate response in adult attention-deficit/hyperactivity disorder patients: results from a naturalistic study. J Neural Transm 123, 859–865. https://doi.org/10.1007/s00702-016-1540-7

Lam, M., Awasthi, S., Watson, H.J., Goldstein, J., Panagiotaropoulou, G., Trubetskoy, V., Karlsson, R., Frei, O., Fan, C.-C., De Witte, W., Mota, N.R., Mullins, N., Brügger, K., Lee, S.H., Wray, N.R., Skarabis, N., Huang, H., Neale, B., Daly, M.J., Mattheisen, M., Walters, R., Ripke, S., 2020. RICOPILI: Rapid Imputation for COnsortias PIpeLIne. Bioinformatics 36, 930–933. https://doi.org/10.1093/bioinformatics/btz633

Marees, A.T., De Kluiver, H., Stringer, S., Vorspan, F., Curis, E., Marie‐Claire, C., Derks, E.M., 2018. A tutorial on conducting genome‐wide association studies: Quality control and statistical analysis. Int J Methods Psych Res 27, e1608. https://doi.org/10.1002/mpr.1608

Ramos, J.K.N., Grevet, E.H., Junger-Santos, I., Ciochetti, N.P., Bandeira, C.E., De Araujo Tavares, M.E., De Oliveira, V.F., Vitola, E.S., Rohde, L.A., Grassi-Oliveira, R., Da Silva, B.S., Bau, C.H.D., Rovaris, D.L., 2025. Shared biological pathways linking ADHD and cortisol variability are related to externalizing behaviors. Psychoneuroendocrinology 181, 107587. https://doi.org/10.1016/j.psyneuen.2025.107587

Victor, M.M., Grevet, E.H., Salgado, C.A.I., Silva, K.L., Sousa, N.O., Karam, R.G., Vitola, E.S., Picon, F.A., Zeni, G.D., Contini, V., Rohde, L.A.P., Belmonte-de-Abreu, P., Bau, C.H.D., 2009. Reasons for Pretreatment Attrition and Dropout From Methylphenidate in Adults With Attention-Deficit/Hyperactivity Disorder: The Role of Comorbidities. Journal of Clinical Psychopharmacology 29, 614–616. https://doi.org/10.1097/JCP.0b013e3181c00b1e

Victor, M.M., Rovaris, D.L., Salgado, C.A.I., Silva, K.L., Karam, R.G., Vitola, E.S., Picon, F.A., Contini, V., Guimarães-da-Silva, P.O., Blaya-Rocha, P., Belmonte-de-Abreu, P.S., Rohde, L.A., Grevet, E.H., Bau, C.H.D., 2014. Severity But Not Comorbidities Predicts Response to Methylphenidate in Adults With Attention-Deficit/Hyperactivity Disorder: Results From a Naturalistic Study. Journal of Clinical Psychopharmacology 34, 212–217. https://doi.org/10.1097/JCP.0000000000000091
