# Supplementary material for: A multi-cohort assessment of the polygenic prediction in ADHD treatment response
Source: Psychiatry Res. Author manuscript; Available in PMC 2026 Jul 7. (PMC13340436; doi:10.1016/j.psychres.2026.116988)

ADHD Model 1

Study	N Resp	N Non−resp	Mean(SE) Resp	Mean(SE) Non−resp	P−value	Odds Ratio [95% CI]
Brazil 1	103	24	0.33 (0.87)	0.41 (0.95)	0.775	1.09 [0.62, 1.92]
Brazil 2	111	25	−0.27 (1.07)	0.06 (0.86)	0.401	1.22 [0.77, 1.94]
Spain	189	43	0.01 (1.04)	−0.06 (0.83)	0.927	0.98 [0.70, 1.38]
Norway 1	249	52	0.14 (0.90)	0.20 (1.08)	0.418	1.14 [0.83, 1.57]
Norway 2	185	17	−0.21 (1.10)	−0.19 (0.81)	0.938	1.02 [0.64, 1.62]
Fixed−Effect Model						1.08 [0.90, 1.29]

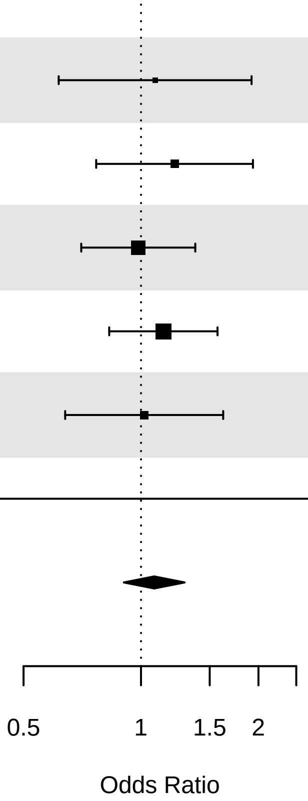

ADHD Model 2

Study	N Resp	N Non−resp	Mean(SE) Resp	Mean(SE) Non−resp	P−value	Odds Ratio [95% CI]
Brazil 1	103	24	0.33 (0.87)	0.41 (0.95)	0.898	1.04 [0.55, 1.97]
Brazil 2	111	25	−0.27 (1.07)	0.06 (0.86)	0.294	1.30 [0.80, 2.12]
Spain	189	43	0.01 (1.04)	−0.06 (0.83)	0.425	0.85 [0.56, 1.28]
Norway 1	236	52	0.13 (0.92)	0.20 (1.08)	0.277	1.20 [0.86, 1.66]
Norway 2	176	15	−0.22 (1.11)	−0.24 (0.78)	0.973	0.99 [0.59, 1.68]
Fixed−Effect Model						1.08 [0.88, 1.31]

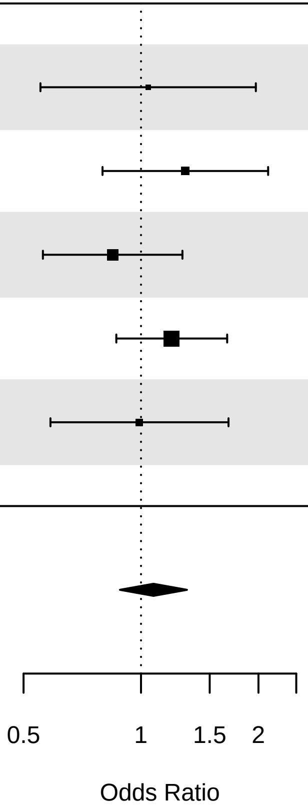

ASD Model 1

Study	N Resp	N Non−resp	Mean(SE) Resp	Mean(SE) Non−resp	P−value	Odds Ratio [95% CI]
Brazil 1	103	24	0.38 (1.05)	0.49 (1.04)	0.653	0.89 [0.53, 1.48]
Brazil 2	111	25	−0.21 (0.94)	0.01 (0.89)	0.421	1.22 [0.75, 2.00]
Spain	189	44	0.03 (0.98)	−0.11 (1.09)	0.347	0.85 [0.60, 1.20]
Norway 1	249	52	−0.54 (0.67)	−0.64 (0.65)	0.368	0.81 [0.51, 1.29]
Norway 2	185	17	0.82 (0.81)	0.96 (1.00)	0.524	1.21 [0.67, 2.20]
Fixed−Effect Model						0.94 [0.77, 1.15]

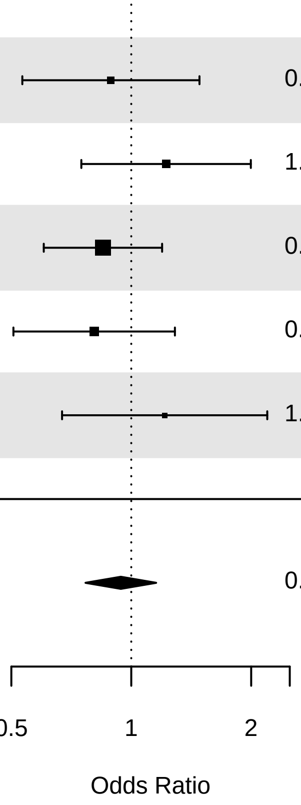

ASD Model 2

Study	N Resp	N Non−resp	Mean(SE) Resp	Mean(SE) Non−resp	P−value	Odds Ratio [95% CI]
Brazil 1	103	24	0.38 (1.05)	0.49 (1.04)	0.548	0.83 [0.46, 1.51]
Brazil 2	111	25	−0.21 (0.94)	0.01 (0.89)	0.484	1.22 [0.70, 2.12]
Spain	189	44	0.03 (0.98)	−0.11 (1.09)	0.891	0.97 [0.64, 1.46]
Norway 1	236	52	−0.56 (0.67)	−0.64 (0.65)	0.465	0.84 [0.52, 1.35]
Norway 2	176	15	0.81 (0.82)	0.89 (1.00)	0.497	1.24 [0.66, 2.32]
Fixed−Effect Model						0.99 [0.78, 1.24]

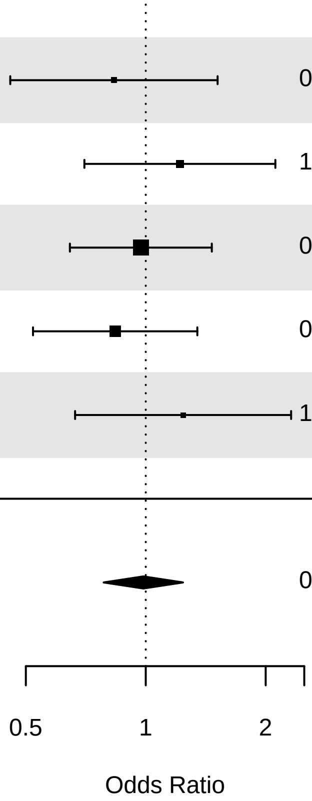

BD Model 1

Study	N Resp	N Non−resp	Mean(SE) Resp	Mean(SE) Non−resp	P−value	Odds Ratio [95% CI]
Brazil 1	103	24	0.49 (0.89)	0.52 (0.99)	0.742	0.91 [0.51, 1.61]
Brazil 2	111	25	−0.37 (0.82)	−0.23 (0.92)	0.984	0.99 [0.55, 1.79]
Spain	189	44	0.02 (1.00)	−0.07 (1.03)	0.471	0.88 [0.63, 1.24]
Norway 1	249	52	0.73 (0.36)	0.79 (0.40)	0.400	1.42 [0.62, 3.24]
Norway 2	185	17	−1.10 (0.52)	−0.10 (0.34)	0.831	0.90 [0.33, 2.41]
Fixed−Effect Model						0.94 [0.74, 1.20]

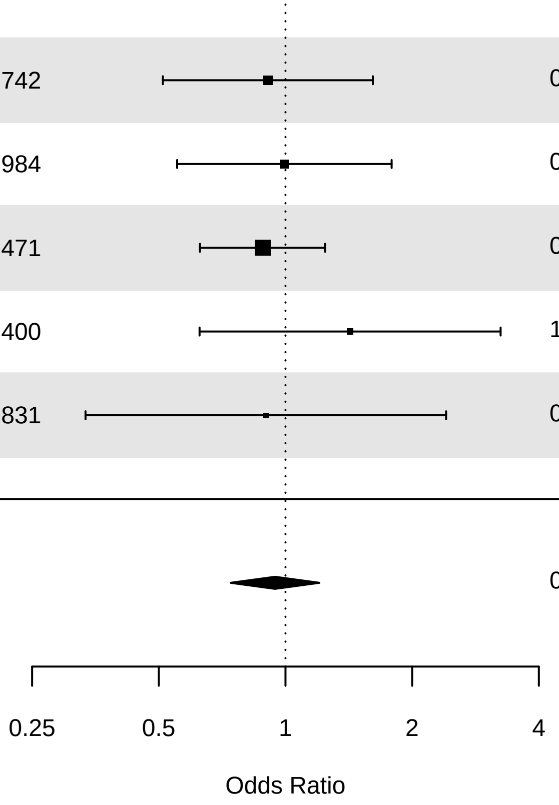

BD Model 2

Study	N Resp	N Non−resp	Mean(SE) Resp	Mean(SE) Non−resp	P−value	Odds Ratio [95% CI]
Brazil 1	103	24	0.49 (0.89)	0.52 (0.99)	0.322	0.71 [0.36, 1.40]
Brazil 2	111	25	−0.37 (0.82)	−0.23 (0.92)	0.894	1.04 [0.54, 2.01]
Spain	189	44	0.02 (1.00)	−0.07 (1.03)	0.516	0.87 [0.57, 1.33]
Norway 1	236	52	0.72 (0.36)	0.79 (0.40)	0.321	1.53 [0.66, 3.56]
Norway 2	176	15	−1.11 (0.53)	−1.07 (0.34)	0.894	1.07 [0.37, 3.08]
Fixed−Effect Model						0.94 [0.71, 1.25]

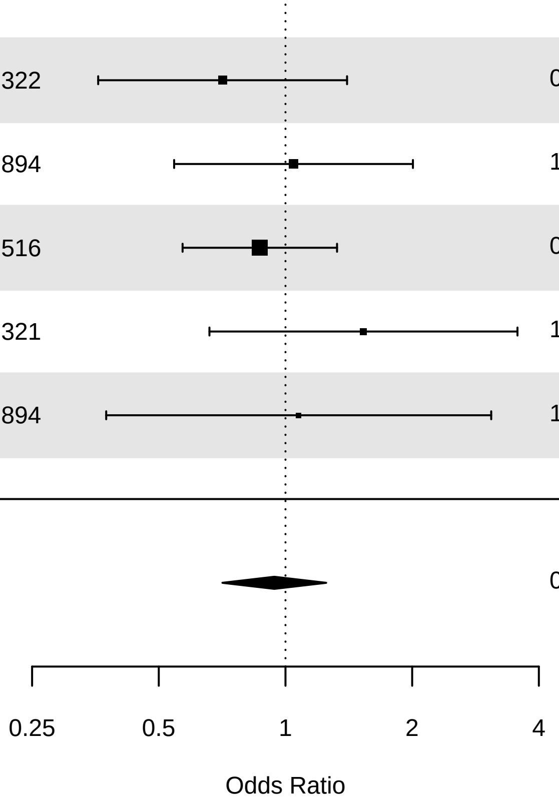

EA Model 1

Study	N Resp	N Non−resp	Mean(SE) Resp	Mean(SE) Non−resp	P−value	Odds Ratio [95% CI]
Brazil 1	103	24	−0.18 (1.09)	0.06 (0.82)	0.217	1.35 [0.84, 2.19]
Brazil 2	111	25	0.27 (0.93)	0.31 (0.94)	0.802	1.06 [0.65, 1.73]
Spain	189	44	0.04 (1.03)	−0.15 (0.87)	0.150	0.78 [0.55, 1.10]
Norway 1	249	52	−0.68 (0.55)	−0.53 (0.53)	0.063	1.77 [0.97, 3.22]
Norway 2	185	17	0.98 (0.69)	0.97 (0.59)	0.821	0.91 [0.42, 1.99]
Fixed−Effect Model						1.04 [0.84, 1.29]

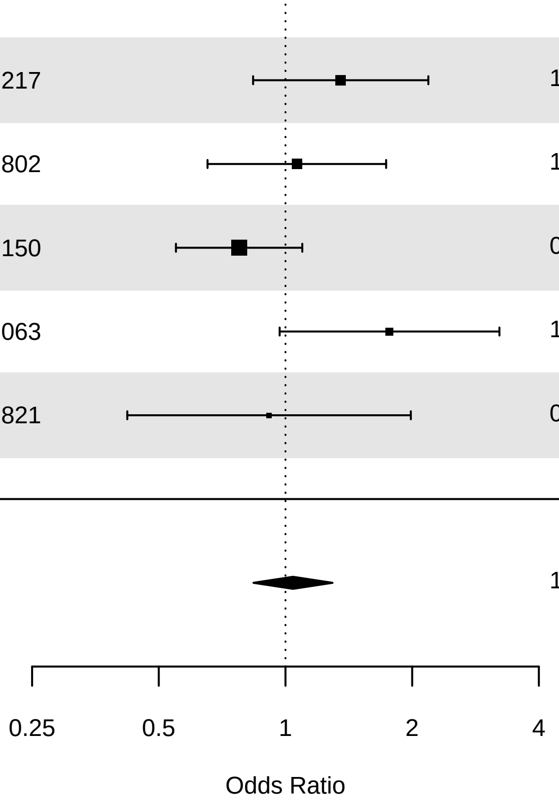

EA Model 2

Study	N Resp	N Non−resp	Mean(SE) Resp	Mean(SE) Non−resp	P−value	Odds Ratio [95% CI]
Brazil 1	103	24	−0.18 (1.09)	0.06 (0.82)	0.235	1.38 [0.81, 2.36]
Brazil 2	111	25	0.27 (0.93)	0.31 (0.94)	0.623	0.87 [0.51, 1.49]
Spain	189	44	0.04 (1.03)	−0.15 (0.87)	0.818	1.05 [0.68, 1.63]
Norway 1	236	52	−0.69 (0.55)	−0.53 (0.53)	0.084	1.70 [0.93, 3.09]
Norway 2	176	15	1.00 (0.70)	1.05 (0.56)	0.815	1.10 [0.48, 2.51]
Fixed−Effect Model						1.17 [0.91, 1.49]

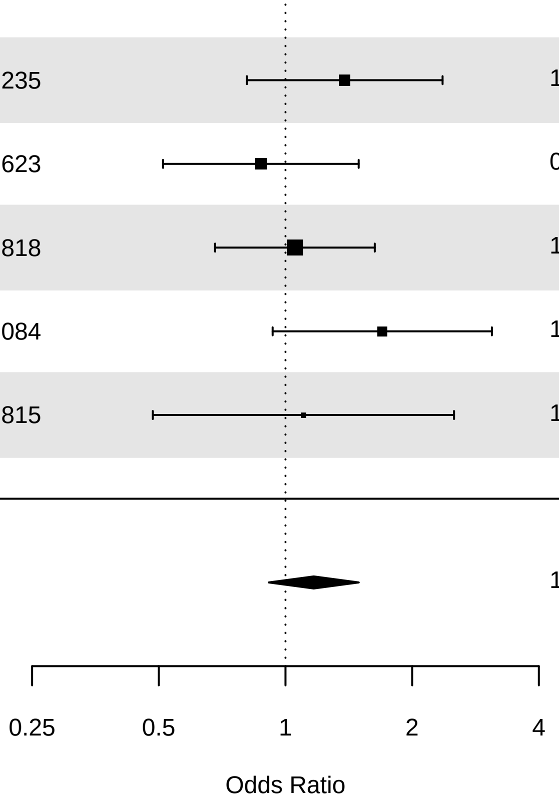

MDD Model 1

Study	N Resp	N Non−resp	Mean(SE) Resp	Mean(SE) Non−resp	P−value	Odds Ratio [95% CI]
Brazil 1	103	24	0.14 (1.03)	0.22 (1.00)	0.805	1.06 [0.67, 1.68]
Brazil 2	111	25	−0.14 (1.00)	−0.15 (1.06)	0.714	0.92 [0.57, 1.47]
Spain	189	44	−0.01 (1.02)	0.05 (0.93)	0.664	1.08 [0.77, 1.52]
Norway 1	249	52	−0.70 (0.51)	−0.61 (0.47)	0.253	1.45 [0.77, 2.72]
Norway 2	185	17	1.04 (0.62)	0.95 (0.60)	0.502	0.74 [0.30, 1.79]
Fixed−Effect Model						1.05 [0.85, 1.30]

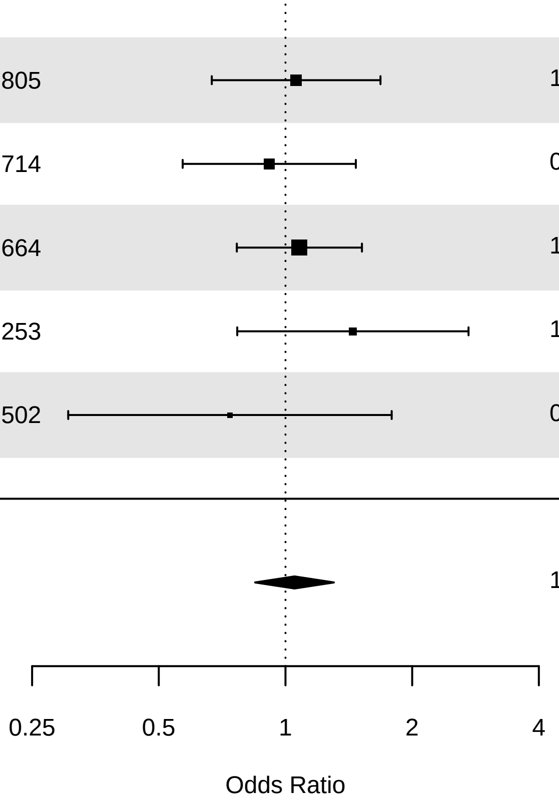

MDD Model 2

Study	N Resp	N Non−resp	Mean(SE) Resp	Mean(SE) Non−resp	P−value	Odds Ratio [95% CI]
Brazil 1	103	24	0.14 (1.03)	0.22 (1.00)	0.636	0.88 [0.51, 1.51]
Brazil 2	111	25	−0.14 (1.00)	−0.15 (1.06)	0.989	1.00 [0.58, 1.72]
Spain	189	44	−0.01 (1.02)	0.05 (0.93)	0.908	0.98 [0.64, 1.48]
Norway 1	236	52	−0.71 (0.51)	−0.61 (0.47)	0.287	1.41 [0.75, 2.66]
Norway 2	176	15	1.04 (0.62)	1.05 (0.56)	0.978	0.99 [0.40, 2.46]
Fixed−Effect Model						1.02 [0.79, 1.30]

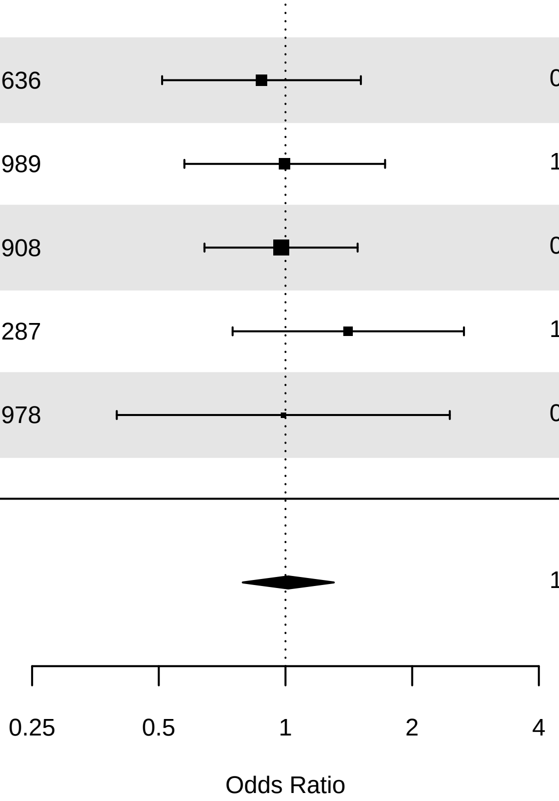

NEU Model 1

Study	N Resp	N Non−resp	Mean(SE) Resp	Mean(SE) Non−resp	P−value	Odds Ratio [95% CI]
Brazil 1	103	24	0.04 (0.94)	−0.00 (1.11)	0.711	1.10 [0.67, 1.81]
Brazil 2	111	25	−0.05 (0.99)	0.41 (0.85)	0.042	1.67 [1.02, 2.73]
Spain	189	44	0.02 (1.00)	−0.09 (1.00)	0.692	0.93 [0.66, 1.31]
Norway 1	249	52	−0.26 (0.79)	−0.34 (0.95)	0.428	0.86 [0.59, 1.25]
Norway 2	185	17	0.46 (1.04)	0.15 (0.88)	0.229	0.73 [0.44, 1.22]
Fixed−Effect Model						0.99 [0.82, 1.19]

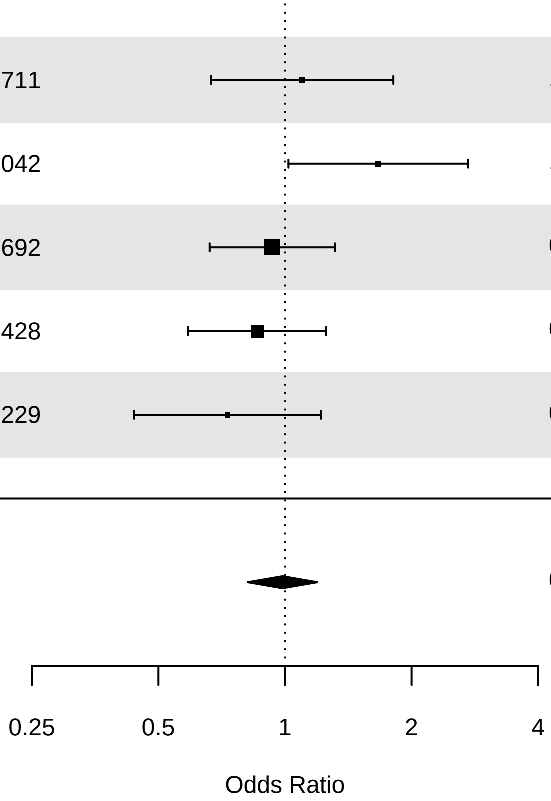

NEU Model 2

Study	N Resp	N Non−resp	Mean(SE) Resp	Mean(SE) Non−resp	P−value	Odds Ratio [95% CI]
Brazil 1	103	24	0.04 (0.94)	−0.00 (1.11)	0.865	1.05 [0.59, 1.87]
Brazil 2	111	25	−0.05 (0.99)	0.41 (0.85)	0.027	1.94 [1.08, 3.47]
Spain	189	44	0.02 (1.00)	−0.09 (1.00)	0.088	0.68 [0.44, 1.06]
Norway 1	236	52	−0.25 (0.80)	−0.34 (0.95)	0.286	0.81 [0.56, 1.19]
Norway 2	176	15	0.44 (1.05)	0.13 (0.91)	0.196	0.69 [0.40, 1.21]
Fixed−Effect Model						0.89 [0.72, 1.10]

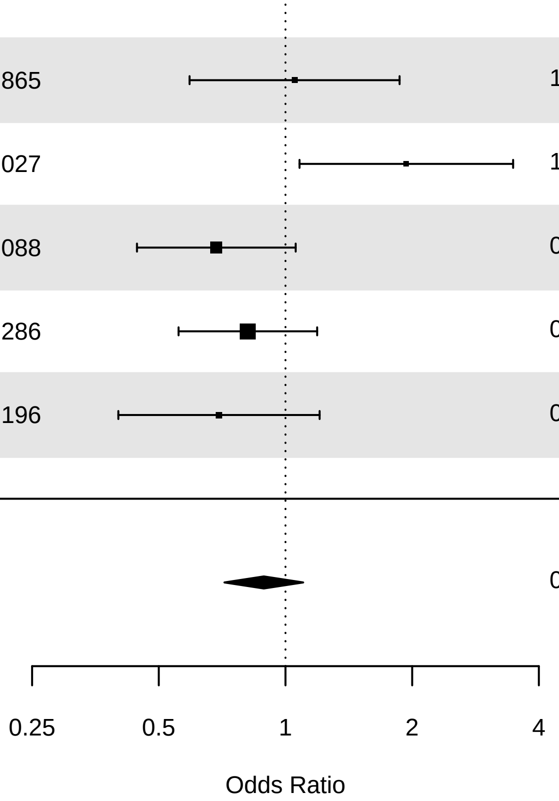

SCZ Model 1

Study	N Resp	N Non−resp	Mean(SE) Resp	Mean(SE) Non−resp	P−value	Odds Ratio [95% CI]
Brazil 1	103	24	0.40 (0.99)	0.39 (0.97)	0.672	0.89 [0.53, 1.50]
Brazil 2	111	25	−0.18 (0.94)	−0.20 (0.89)	0.788	0.93 [0.57, 1.54]
Spain	189	44	0.01 (1.02)	−0.06 (0.91)	0.451	0.88 [0.62, 1.24]
Norway 1	249	52	−0.15 (0.93)	−0.10 (0.97)	0.774	1.05 [0.75, 1.46]
Norway 2	185	17	0.24 (1.05)	−0.31 (1.11)	0.037	0.57 [0.33, 0.96]
Fixed−Effect Model						0.89 [0.74, 1.07]

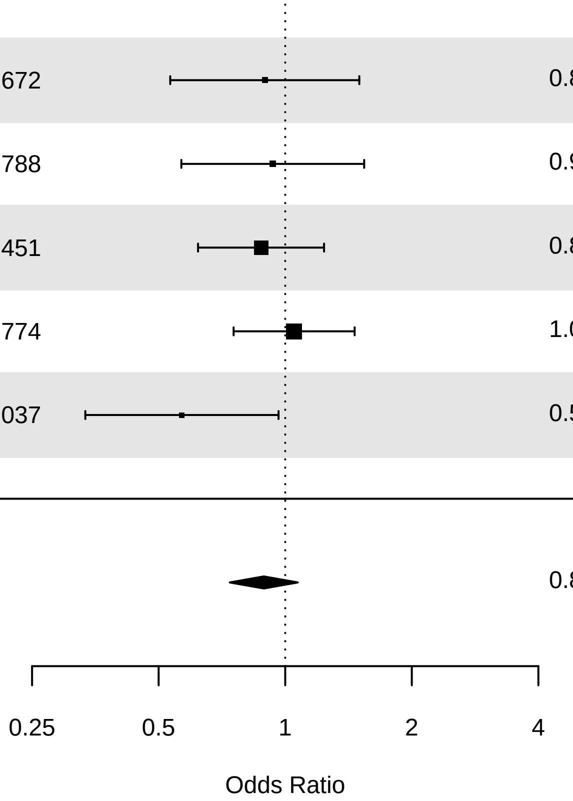

SCZ Model 2

Study	N Resp	N Non−resp	Mean(SE) Resp	Mean(SE) Non−resp	P−value	Odds Ratio [95% CI]
Brazil 1	103	24	0.40 (0.99)	0.39 (0.97)	0.416	0.78 [0.43, 1.42]
Brazil 2	111	25	−0.18 (0.94)	−0.20 (0.89)	0.836	1.06 [0.61, 1.85]
Spain	189	44	0.01 (1.02)	−0.06 (0.91)	0.850	0.96 [0.63, 1.47]
Norway 1	236	52	−0.17 (0.94)	−0.10 (0.97)	0.647	1.08 [0.77, 1.51]
Norway 2	176	15	0.24 (1.06)	−0.21 (1.14)	0.233	0.72 [0.42, 1.23]
Fixed−Effect Model						0.95 [0.78, 1.17]

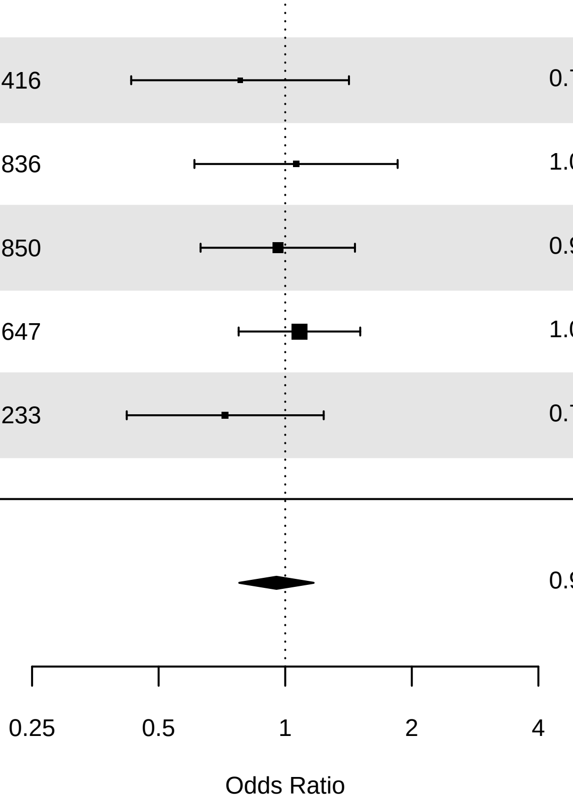

Supplement: 2 [file NIHMS2190217-supplement-2.pdf]
