# Supplementary material for: A multi-cohort assessment of the polygenic prediction in ADHD treatment response
Source: Psychiatry Res. Author manuscript; Available in PMC 2026 Jul 7. (PMC13340436; doi:10.1016/j.psychres.2026.116988)
Supplement: 3 [file NIHMS2190217-supplement-3.pdf]

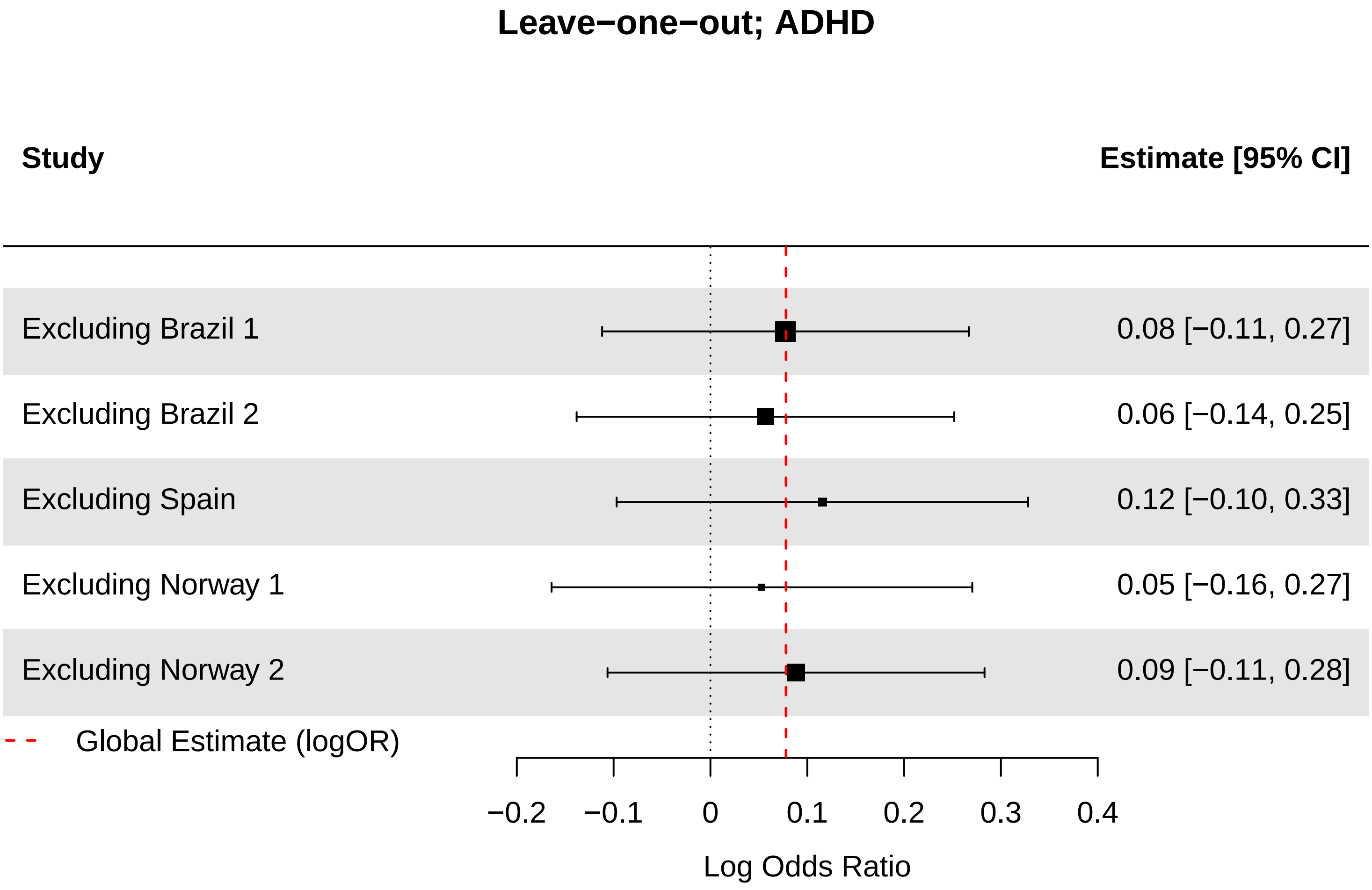
Leave−one−out; ADHD
Study
Estimate [95% CI]
Excluding Brazil 1
0.08 [−0.11, 0.27]
Excluding Brazil 2
0.06 [−0.14, 0.25]
Excluding Spain
0.12 [−0.10, 0.33]
Excluding Norway 1
0.05 [−0.16, 0.27]
Excluding Norway 2
0.09 [−0.11, 0.28]
Global Estimate (logOR)
−0.2
−0.1
0
0.1
0.2
0.3
0.4
Log Odds Ratio

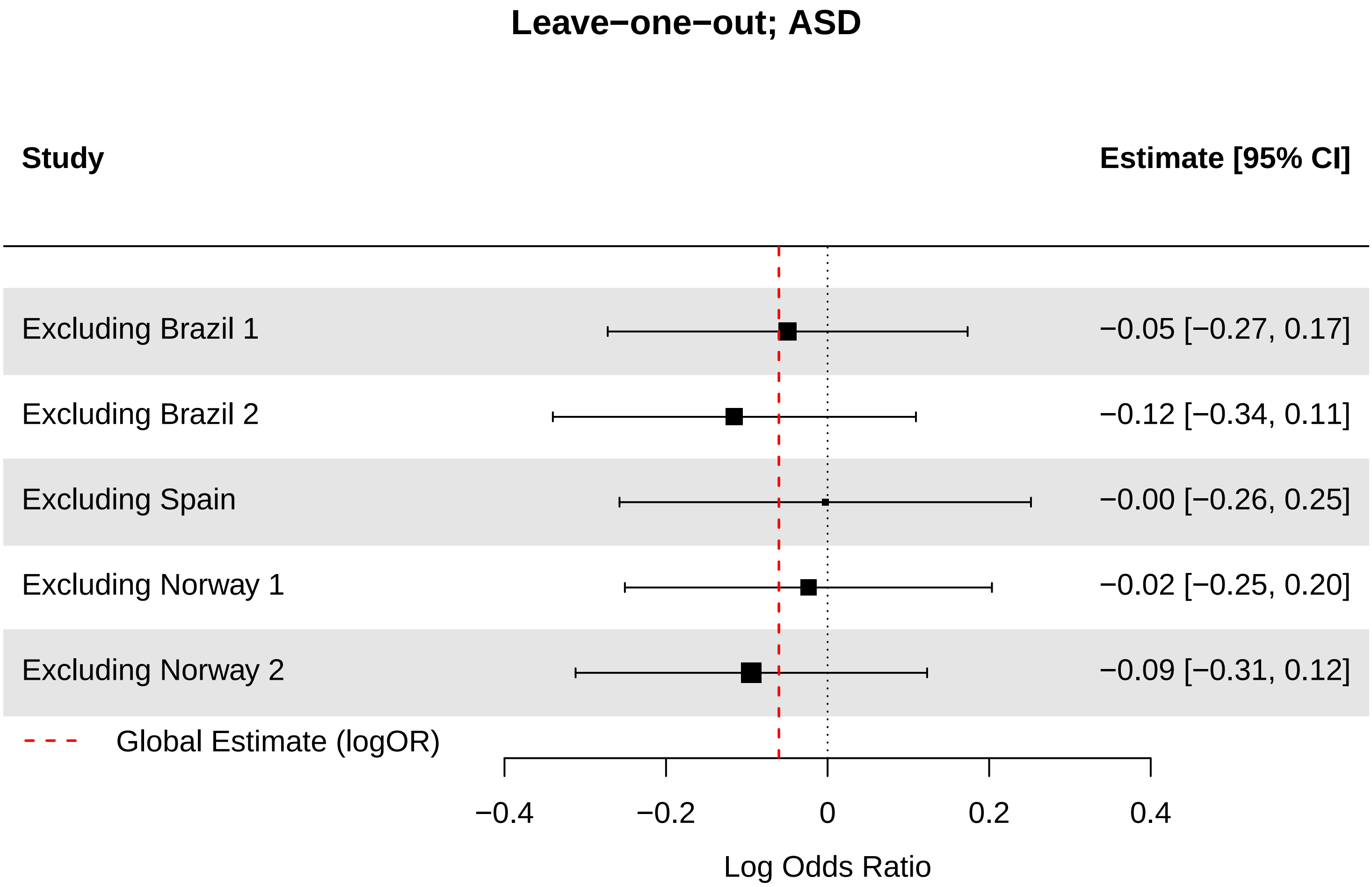
Leave−one−out; ASD
Study
Estimate [95% CI]
Excluding Brazil 1
−0.05 [−0.27, 0.17]
Excluding Brazil 2
−0.12 [−0.34, 0.11]
Excluding Spain
−0.00 [−0.26, 0.25]
Excluding Norway 1
−0.02 [−0.25, 0.20]
Excluding Norway 2
−0.09 [−0.31, 0.12]
Global Estimate (logOR)
−0.4
−0.2
0
0.2
0.4
Log Odds Ratio

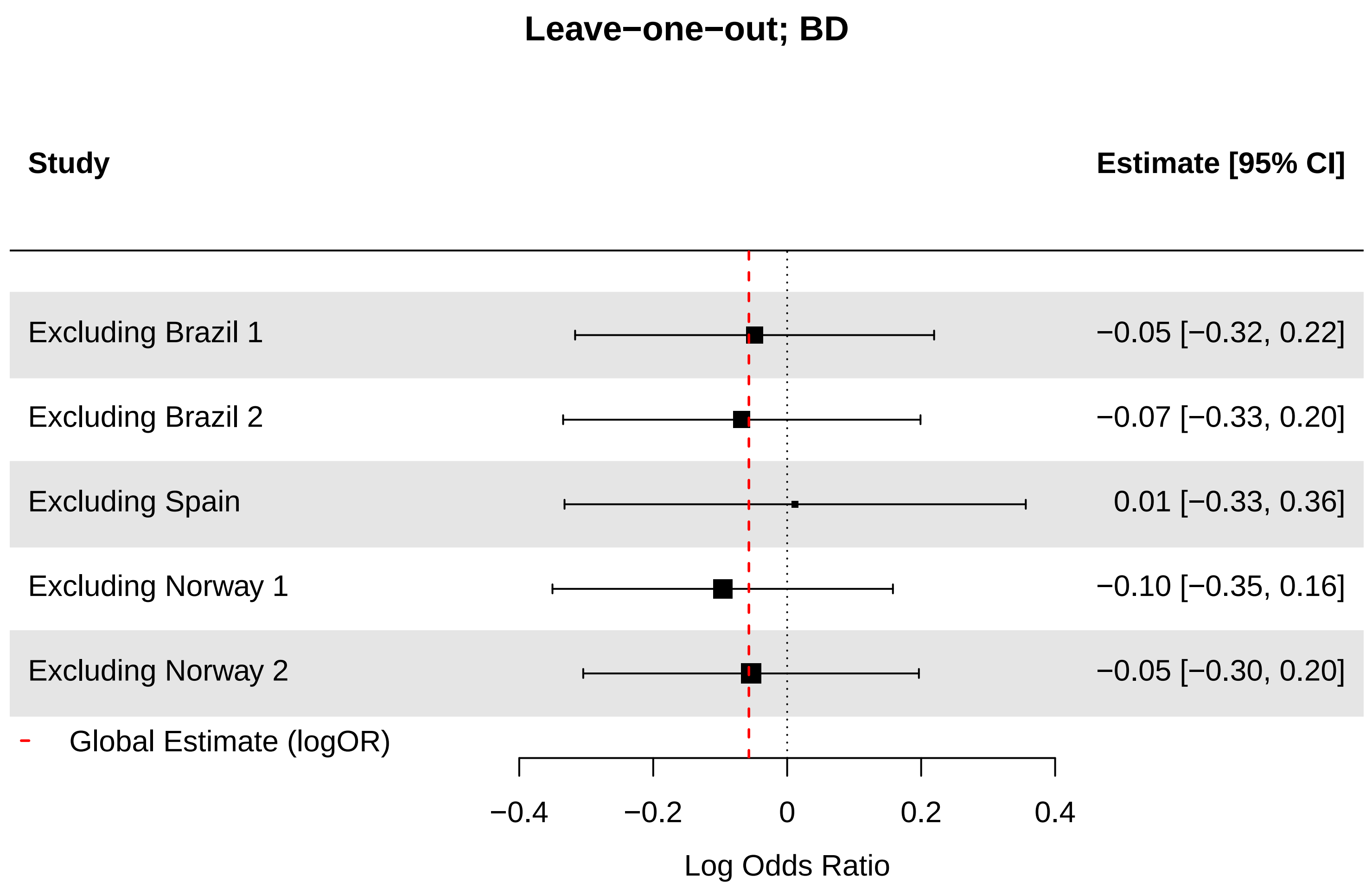
Leave−one−out; BD
Study
Estimate [95% CI]
Excluding Brazil 1
−0.05 [−0.32, 0.22]
Excluding Brazil 2
−0.07 [−0.33, 0.20]
Excluding Spain
0.01 [−0.33, 0.36]
Excluding Norway 1
−0.10 [−0.35, 0.16]
Excluding Norway 2
−0.05 [−0.30, 0.20]
Global Estimate (logOR)
−0.4
−0.2
0
0.2
0.4
Log Odds Ratio

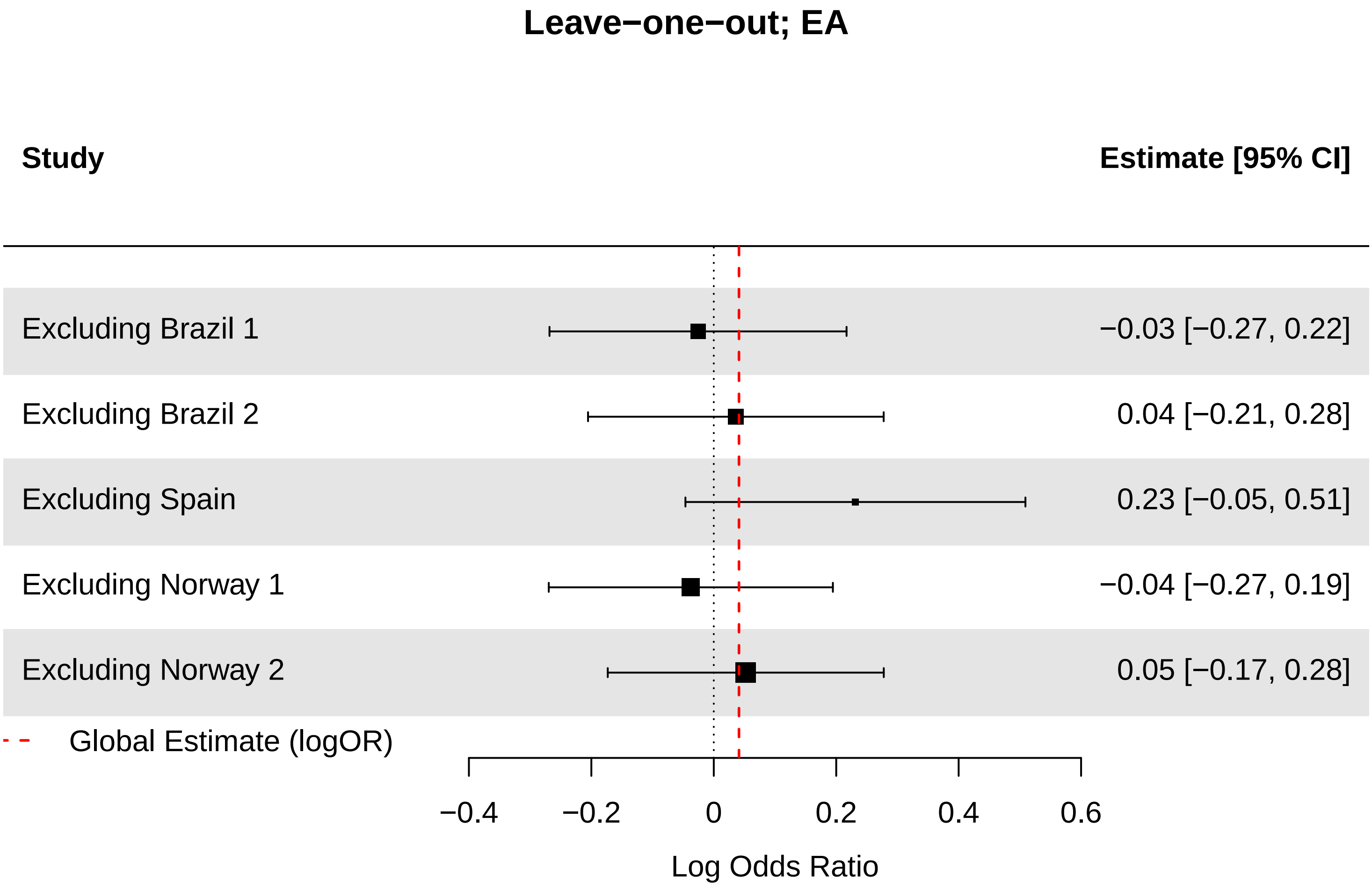
Leave−one−out; EA
Study
Estimate [95% CI]
Excluding Brazil 1
−0.03 [−0.27, 0.22]
Excluding Brazil 2
0.04 [−0.21, 0.28]
Excluding Spain
0.23 [−0.05, 0.51]
Excluding Norway 1
−0.04 [−0.27, 0.19]
Excluding Norway 2
0.05 [−0.17, 0.28]
Global Estimate (logOR)
−0.4
−0.2
0
0.2
0.4
0.6
Log Odds Ratio

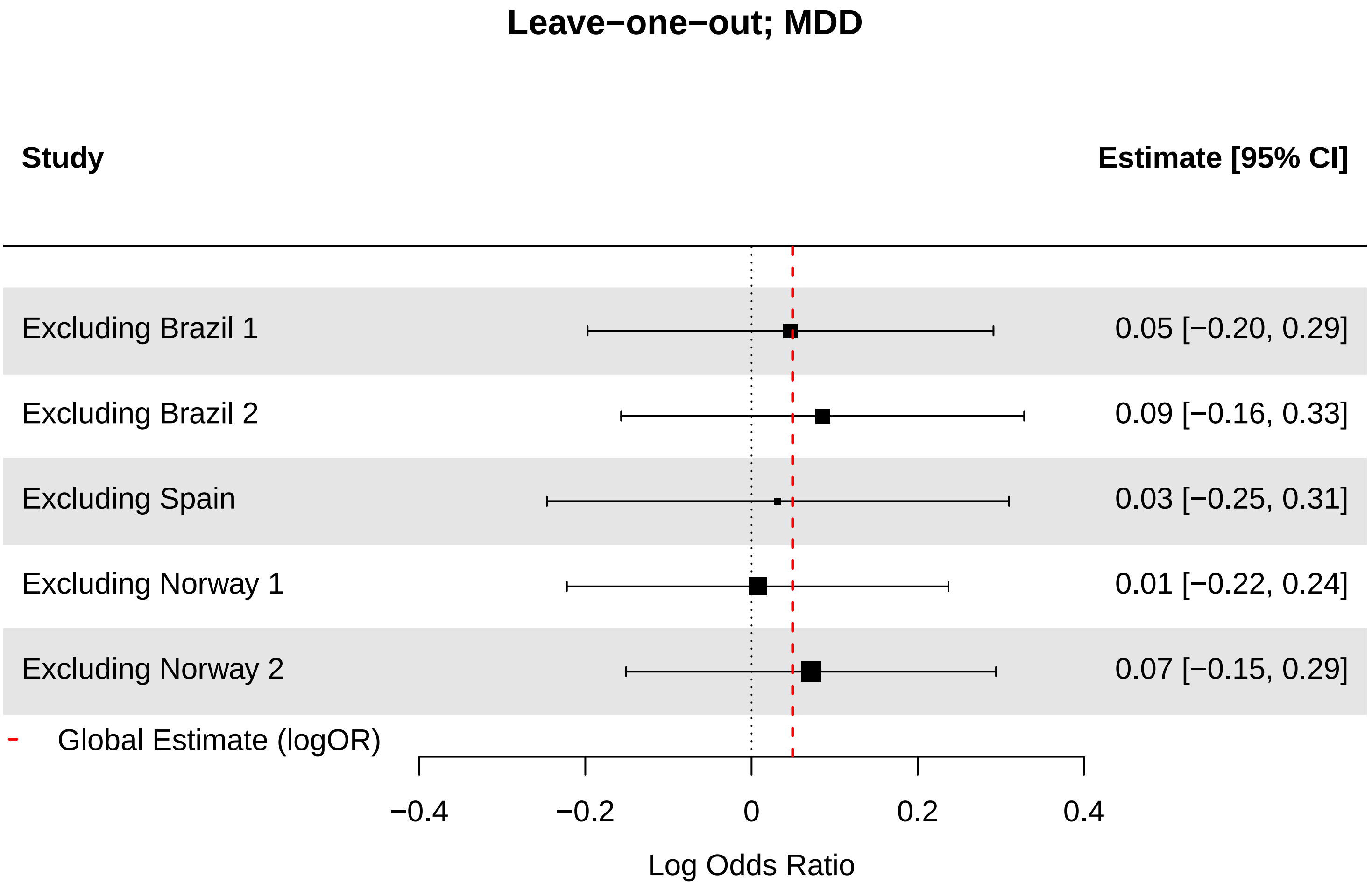
Leave−one−out; MDD
Study
Estimate [95% CI]
Excluding Brazil 1
0.05 [−0.20, 0.29]
Excluding Brazil 2
0.09 [−0.16, 0.33]
Excluding Spain
0.03 [−0.25, 0.31]
Excluding Norway 1
0.01 [−0.22, 0.24]
Excluding Norway 2
0.07 [−0.15, 0.29]
Global Estimate (logOR)
−0.4
−0.2
0
0.2
0.4
Log Odds Ratio

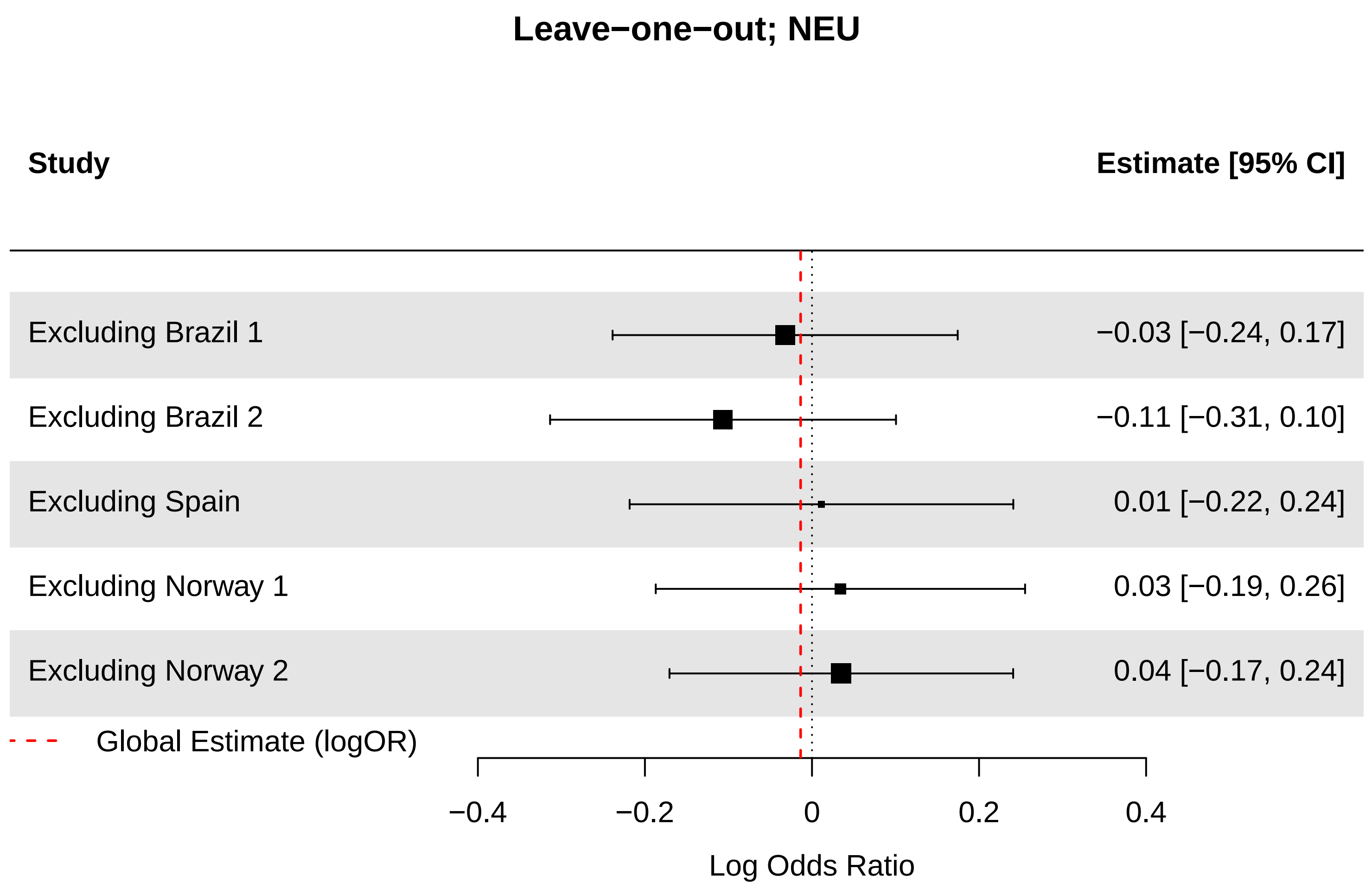
Leave−one−out; NEU
Study
Estimate [95% CI]
Excluding Brazil 1
−0.03 [−0.24, 0.17]
Excluding Brazil 2
−0.11 [−0.31, 0.10]
Excluding Spain
0.01 [−0.22, 0.24]
Excluding Norway 1
0.03 [−0.19, 0.26]
Excluding Norway 2
0.04 [−0.17, 0.24]
Global Estimate (logOR)
−0.4
−0.2
0
0.2
0.4
Log Odds Ratio

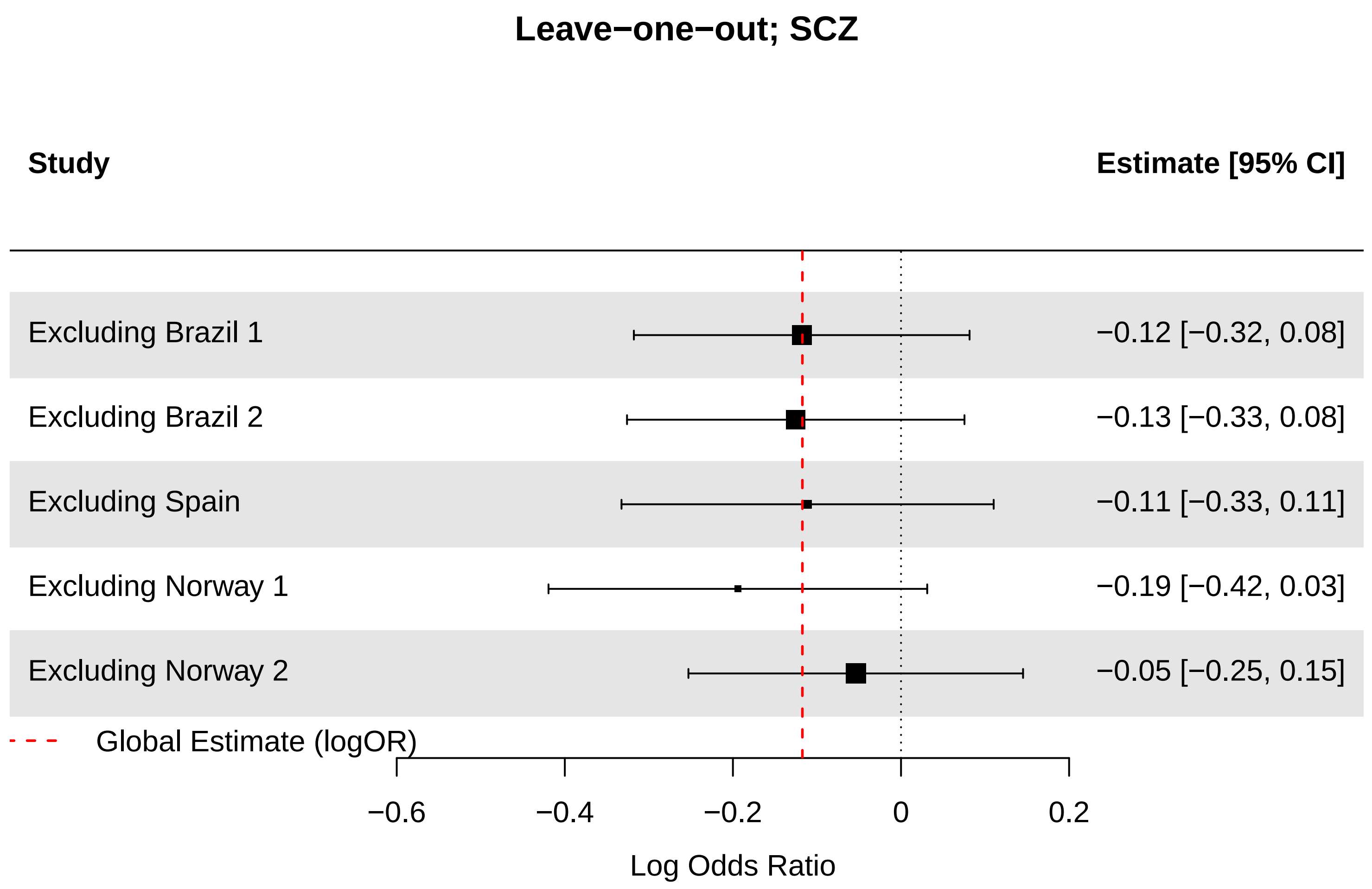
Leave−one−out; SCZ
Study
Estimate [95% CI]
Excluding Brazil 1
−0.12 [−0.32, 0.08]
Excluding Brazil 2
−0.13 [−0.33, 0.08]
Excluding Spain
−0.11 [−0.33, 0.11]
Excluding Norway 1
−0.19 [−0.42, 0.03]
Excluding Norway 2
−0.05 [−0.25, 0.15]
Global Estimate (logOR)
−0.6
−0.4
−0.2
0
0.2
Log Odds Ratio
