# Supplementary figures and images for: A multi-cohort assessment of the polygenic prediction in ADHD treatment response
Source: Psychiatry Res. Author manuscript; Available in PMC 2026 Jul 7. (PMC13340436; doi:10.1016/j.psychres.2026.116988)

# ADHD

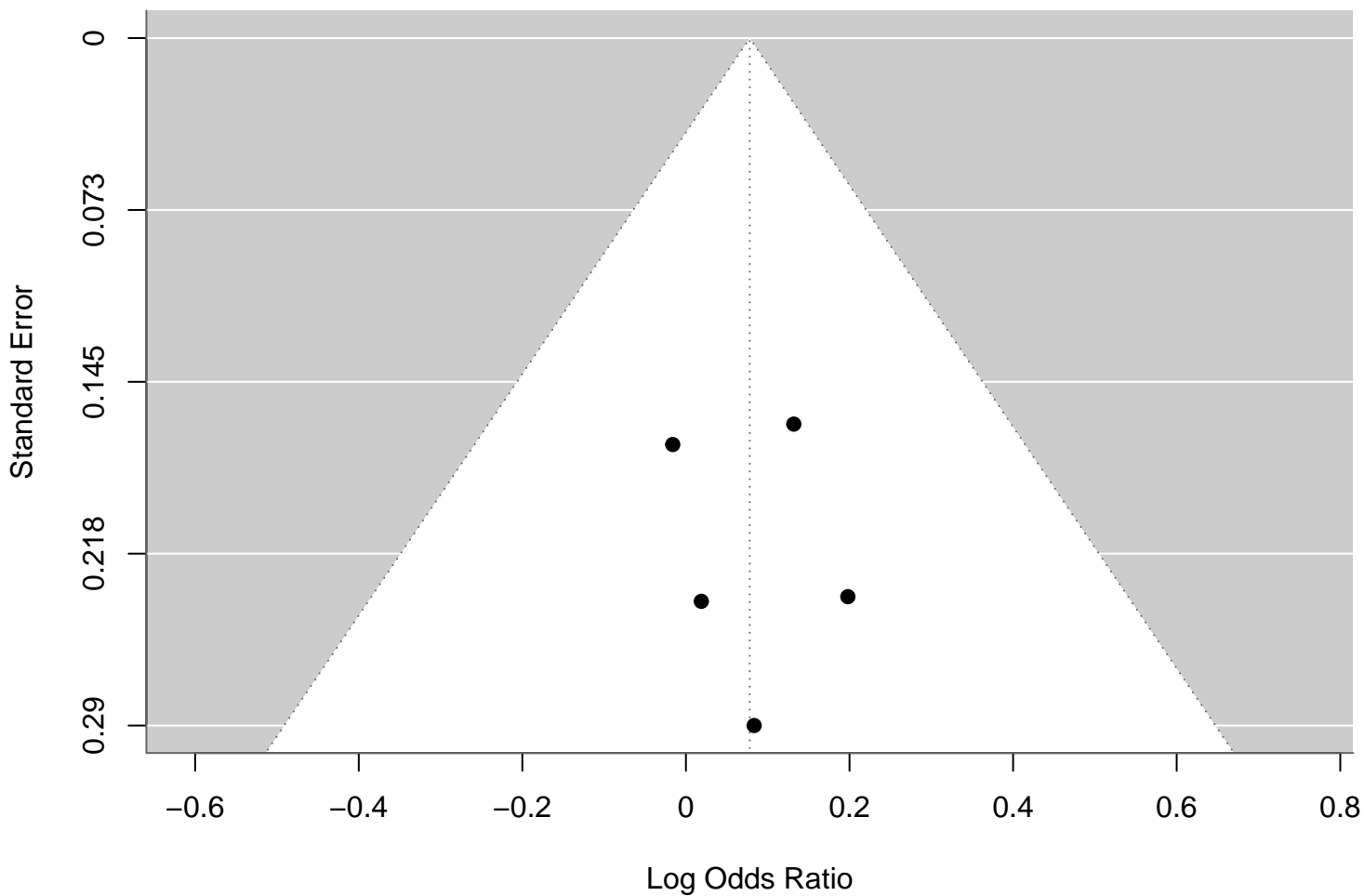

# ASD

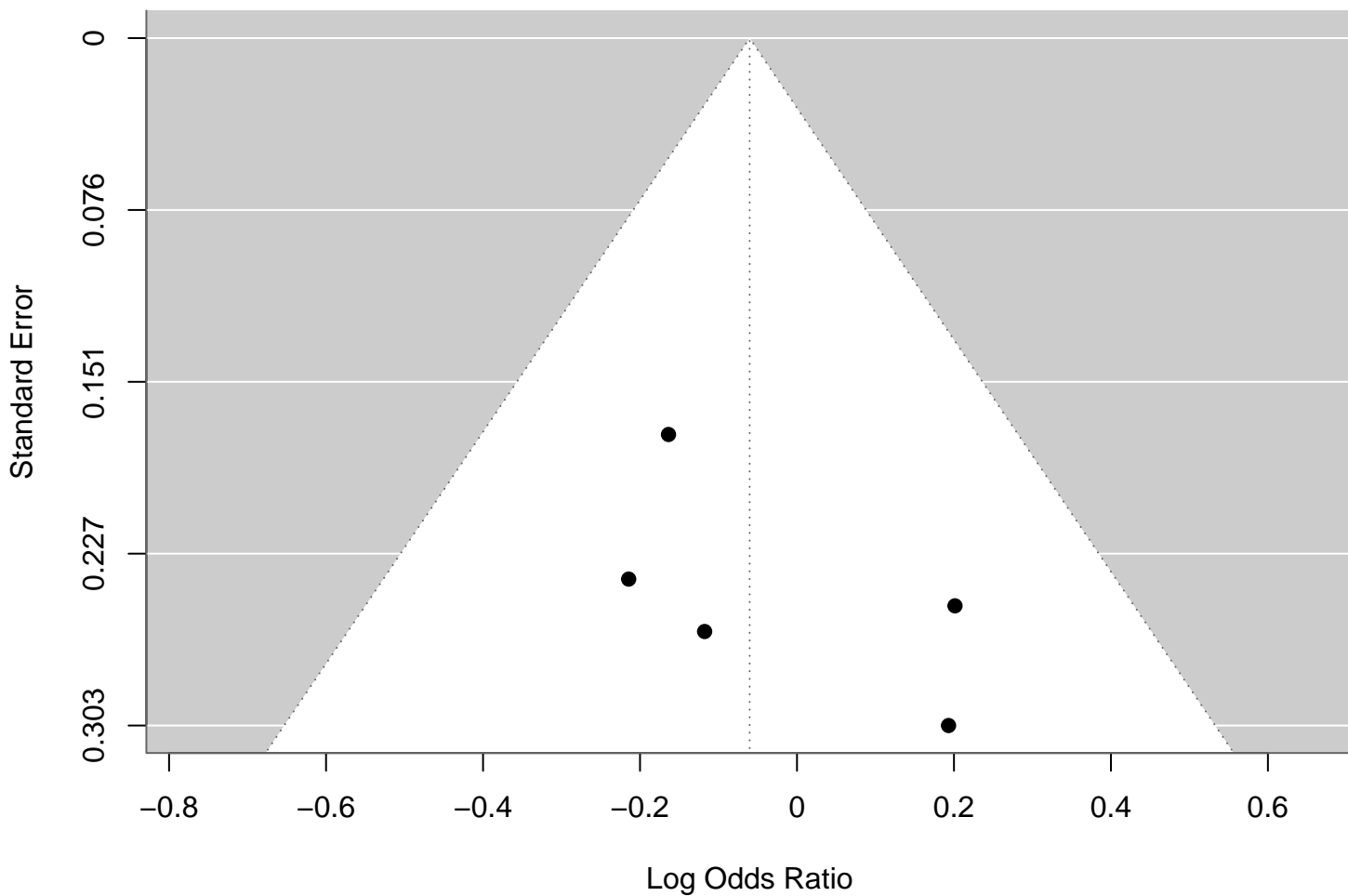

**BD**

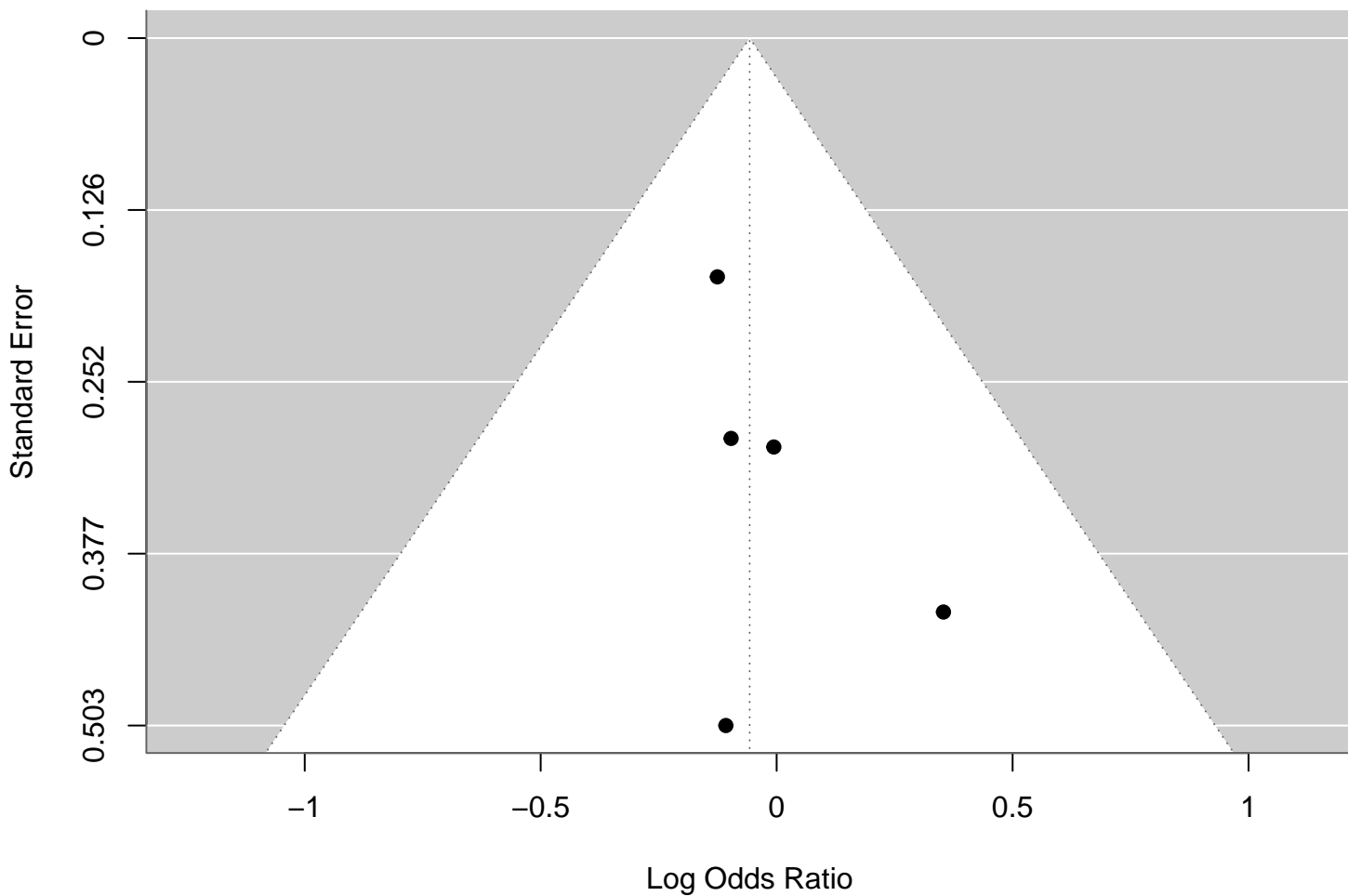

EA

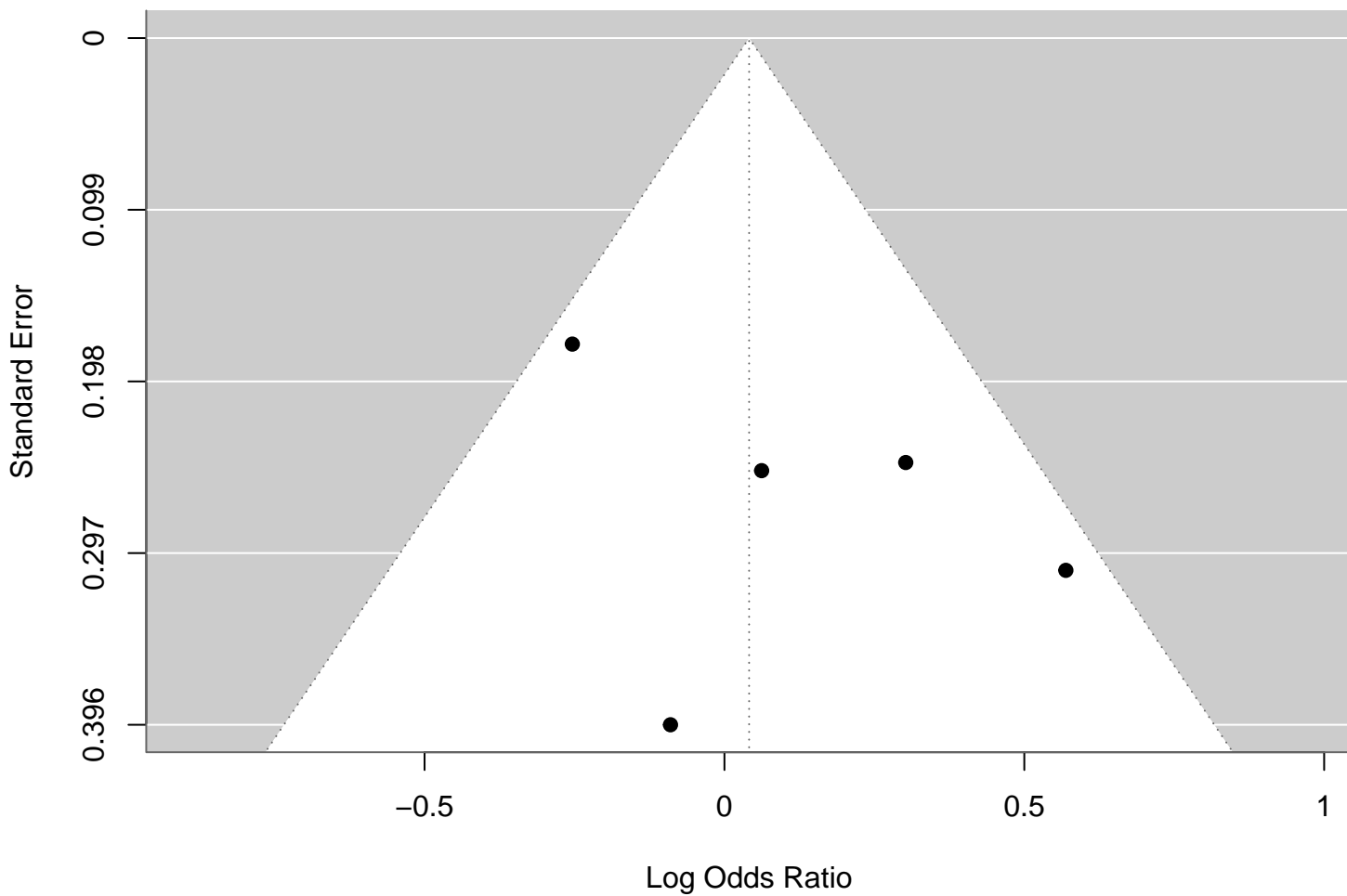

# MDD

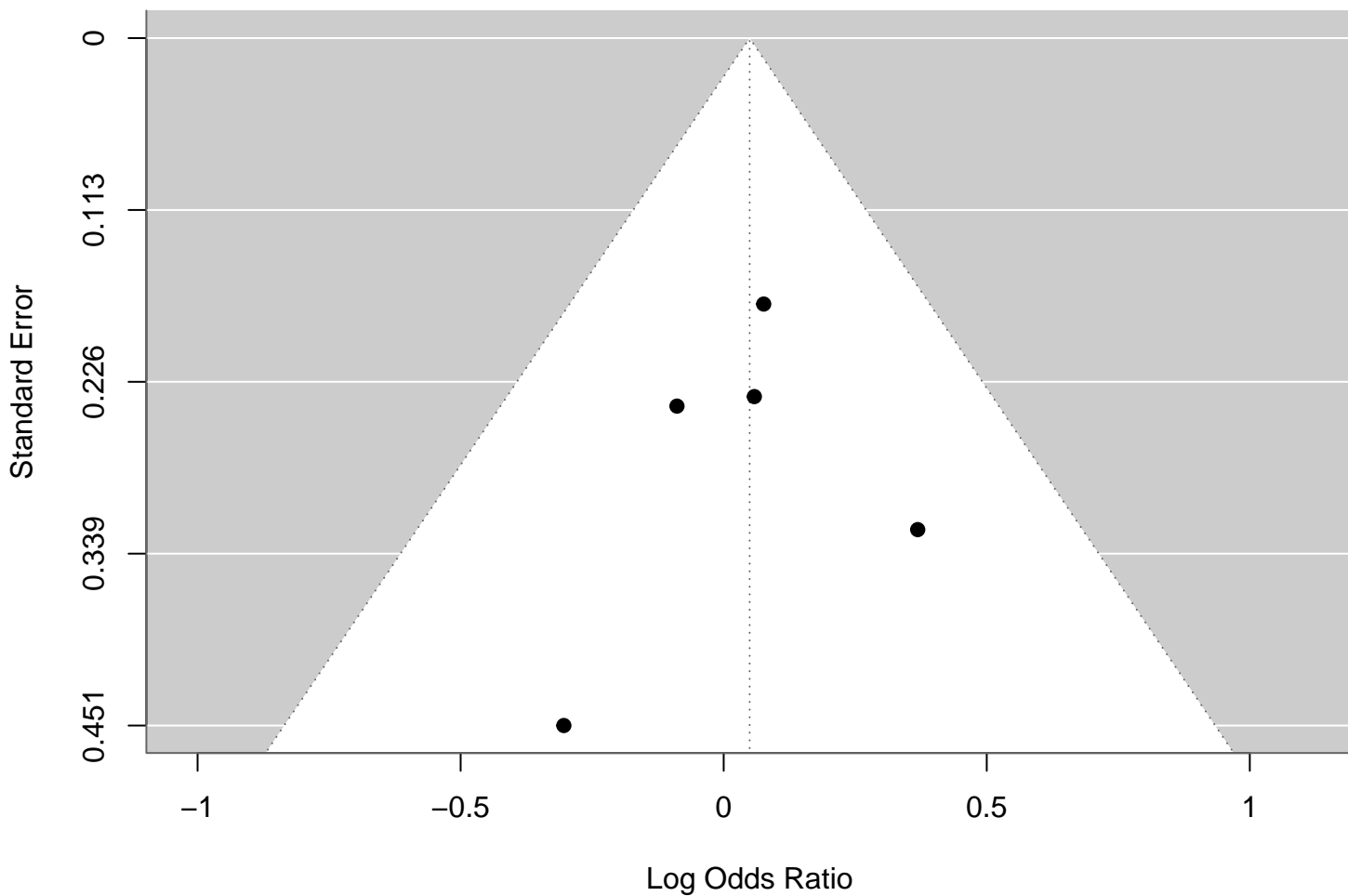

# NEU

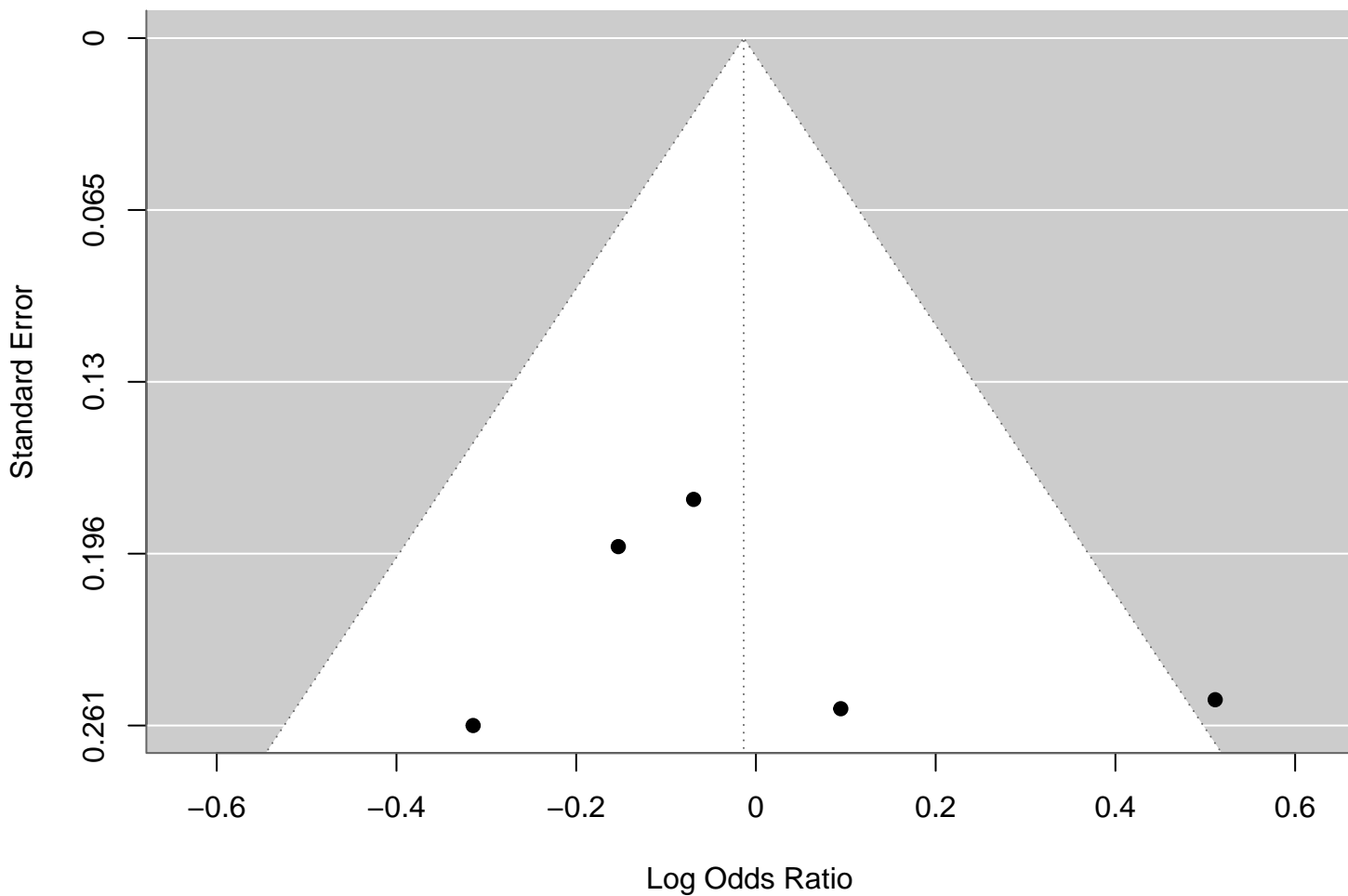

# SCZ

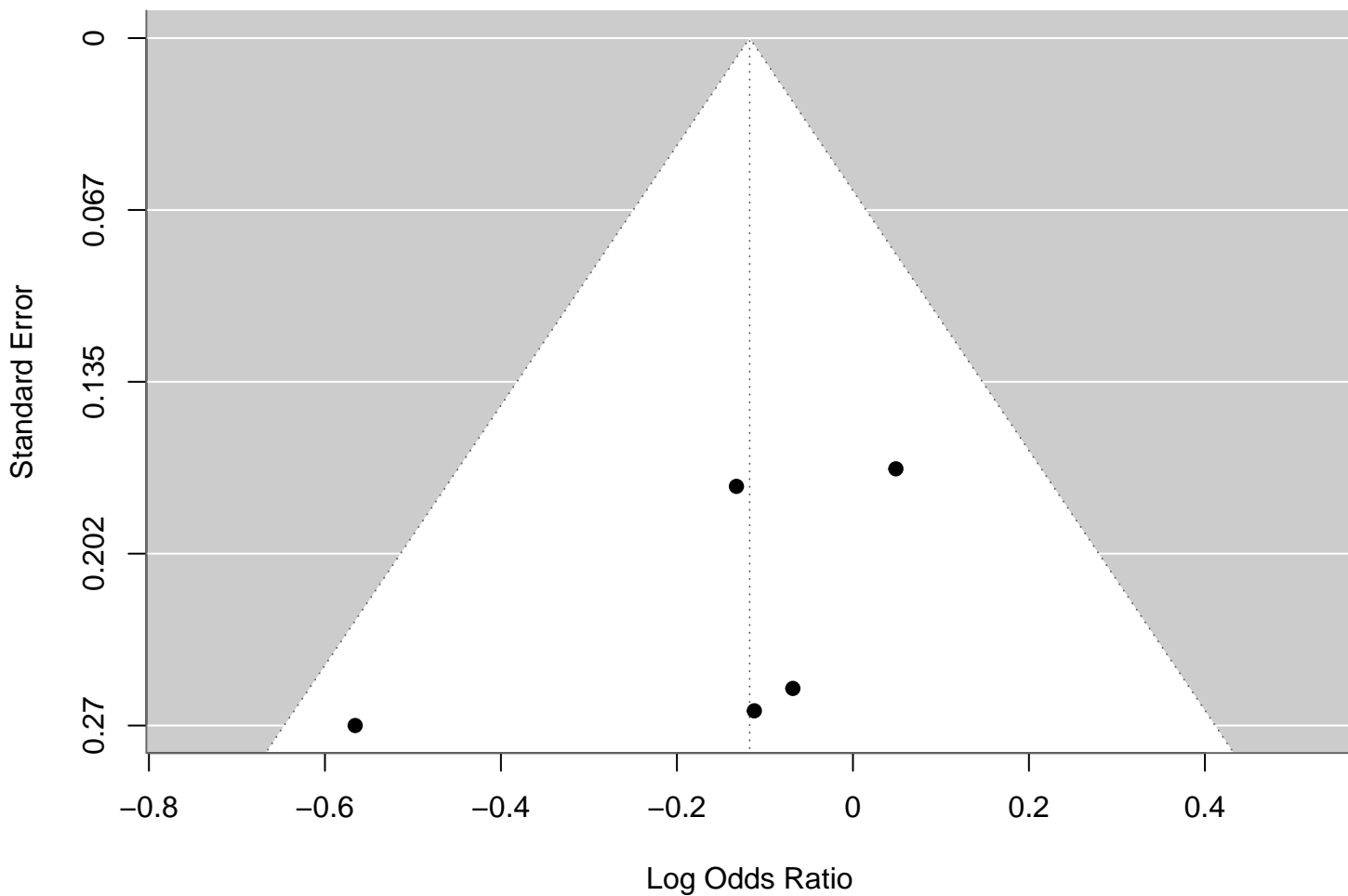

Supplement: 4 [file NIHMS2190217-supplement-4.pdf]
