# Supplementary material for: A multi-cohort assessment of the polygenic prediction in ADHD treatment response
Source: Psychiatry Res. Author manuscript; Available in PMC 2026 Jul 7. (PMC13340436; doi:10.1016/j.psychres.2026.116988)
Supplement: 5 [file NIHMS2190217-supplement-5.docx]

**A multi-cohort assessment of the polygenic prediction in ADHD treatment response**

**SUPPLEMENTARY FIGURE LEGENDS**

**Supplementary Figure S1.** **Forest plots from fixed-effect meta-analyses showing the association between polygenic scores (PGS) and ADHD treatment response across five cohorts.** Displayed are the effect sizes (odds ratios (OR) [95% confidence intervals (CI)] for trait-specific PGS derived from GWAS of ADHD, ASD, BD, EA, MDD, NEU, and SCZ. Study-specific, along with the overall pooled estimates, are shown. OR < 1 indicate higher polygenic liability among treatment responders. Sample sizes and PGS mean (SE) for responders (N Resp) and non-responders (N Non-resp), alongside with p-values are shown for each study. Brazil 1, Brazilian cohort of mixed ancestries; Brazil 2, Brazilian cohort of European ancestry, Spain, Spanish cohort of European ancestry; Norway 1, Norwegian cohort from European ancestry – Broad sample; Norway 2, Norwegian cohort from European ancestry – deCODE sample. Model 1: sex, age, first five principal components were included as covariates; Model 2: for Brazil 1 and Brazil 2, sex, age, baseline symptom severity, concomitant use of psychiatric medications, comorbidity with generalized anxiety disorder and social phobia were included as covariates; for Spain, sex, age, first five principal components, baseline symptom severity, methylphenidate formulation (long or short acting), comorbidity with oppositional defiant disorders were included as covariates; for Norway 1 and 2, sex, age, first five principal components, baseline symptom severity, comorbidity with anxiety or depression were included as covariates. **Abbreviations:** OR, odds ratio; CI, confidence interval; ADHD, attention-deficit/hyperactivity disorder; ASD, autism spectrum disorder; BD, bipolar disorder; EA, educational attainment; MDD, major depressive disorder; NEU, neuroticism; SCZ, schizophrenia.

**Supplementary Figure S2. Leave-one-out meta-analysis forest plots for the association between polygenic scores (PGS) and ADHD treatment response (Model 1).** Each panel shows the meta-analytic effect estimate (log odds ratios, log[OR]) and 95% confidence interval (CI), recalculated after sequentially excluding one cohort at a time. The red dashed line represents the overall global estimate when all cohorts are included. Results are presented for PGS derived from GWAS of ADHD, ASD, BD, EA, MDD, NEU, and SCZ. **Abbreviations:** OR, odds ratio; CI, confidence interval; ADHD, attention-deficit/hyperactivity disorder; ASD, autism spectrum disorder; BD, bipolar disorder; EA, educational attainment; MDD, major depressive disorder; NEU, neuroticism; SCZ, schizophrenia; Brazil 1, Brazilian cohort of mixed ancestries; Brazil 2, Brazilian cohort of European ancestry, Spain, Spanish cohort of European ancestry; Norway 1, Norwegian cohort from European ancestry – Broad sample; Norway 2, Norwegian cohort from European ancestry – deCODE sample.

**Supplementary Figure S3. Funnel plots showing the distribution of study-specific effect sizes (log odds ratios; log[OR]) against their standard errors for the meta-analyses of polygenic score (PGS) associations with ADHD treatment response (Model 1).** Each plot displays one base GWAS phenotype (ADHD, ASD, BD, EA, MDD, NEU, SCZ). The dashed line represents the pooled overall effect size with the pseudo-confidence region. Each black point corresponds to an individual study. Symmetry around the pooled effect provides a visual assessment of potential between-study heterogeneity. In the absence of heterogeneity, the points are expected to form a symmetrical funnel shape, with most falling within the pseudo-confidence region. **Abbreviations:** ADHD, attention-deficit/hyperactivity disorder; ASD, autism spectrum disorder; BD, bipolar disorder; EA, educational attainment; MDD, major depressive disorder; NEU, neuroticism; SCZ, schizophrenia.
